# Utility of MF-non coding region for measles molecular surveillance during post-elimination phase, Spain, 2017–2020

**DOI:** 10.3389/fmicb.2023.1143933

**Published:** 2023-05-22

**Authors:** Camille Jacqueline, Ana María Gavilán, Noemí López-Perea, Ana Raquel Penedos, Josefa Masa-Calles, Juan E. Echevarría, Aurora Fernández-García, on behalf of the MMR Study Group

**Affiliations:** ^1^Centro Nacional de Microbiología, Instituto de Salud Carlos III, Majadahonda, Majadahonda, Spain; ^2^European Public Health Microbiology Training Programme (EUPHEM), European Centre for Disease Prevention and Control (ECDC), Stockholm, Sweden; ^3^CIBER de Epidemiología y Salud Pública (CIBERESP), Madrid, Spain; ^4^Centro Nacional de Epidemiología, Instituto de Salud Carlos III, Madrid, Spain; ^5^United Kingdom Health Security Agency, London, United Kingdom

**Keywords:** phylodynamic analyses, mathematical model, outbreaks, phylogenetic analysis, B3 genotype, D8 genotype

## Abstract

**Background:**

In countries entering the post-elimination phase for measles, the study of variants by sequencing of 450 nucleotides of the N gene (N450) does not always allow the tracing of chains of transmission. Indeed, between 2017 and 2020, most measles virus sequences belonged to either the MVs/Dublin.IRL/8.16 (B3-Dublin) or the MVs/Gir Somnath.IND/42.16 (D8-Gir Somnath) variants. We evaluated the additional use of a non-coding region (MF-NCR) as a tool to enhance resolution and infer case origin, chains of transmission and characterize outbreaks.

**Methods:**

We obtained 115 high-quality MF-NCR sequences from strains collected from Spanish patients infected with either B3-Dublin or D8-Gir Somnath variants between 2017 and 2020, performed epidemiological, phylogenetic and phylodynamic analyses and applied a mathematical model to determine relatedness among identified clades.

**Results:**

Applying this model allowed us to identify phylogenetic clades potentially derived from concomitant importations of the virus rather than single chain of transmission, inferred based on only N450 and epidemiology data. In a third outbreak, we found two related clades that corresponded to two chains of transmission.

**Discussion:**

Our results show the ability of the proposed method to improve identification of simultaneous importations in the same region which could trigger enhanced contact tracing. Moreover, the identification of further transmission chains indicates that the size of import-related outbreaks was smaller than previously found, supporting the interpretation that endemic measles transmission was absent in Spain between 2017 and 2020. We suggest considering the use of the MF-NCR region in conjunction with the study of N450 variants in future WHO recommendations for measles surveillance.

## Introduction

Measles is caused by the measles virus (MeV) and is a major cause of morbidity worldwide. The implementation of the Global Measles and Rubella Strategic Plan 2012–2020 saw a significant reduction in measles incidence, especially through vaccination and improvements in surveillance (Wang et al., 2022). Despite this progress, the elimination targets for 2020 were not met and twice as many cases were reported globally in 2018 compared to 2017 (World Health Organization, 2020a). This upward trend was continuing in 2019 with several countries (e.g., Democratic Republic of the Congo, Ukraine, and Brazil) experiencing large outbreaks, just before the COVID-19 pandemic led to an interruption of routine vaccination in several countries and to fewer case notifications (https://www.cdc.gov/globalhealth/measles/news/covid-impact-on-measles-vaccination.html). Therefore, World Health Organization (2020b) launched a new strategic plan to eliminate measles by 2030.

Since 2014, measles presented a post-elimination profile in Spain with most cases being classified as imported or import-related and predominantly detected in adults as well as frequently associated with healthcare environments (López-Perea et al., 2021). In 2017, WHO declared measles eliminated from Spain, as endemic measles transmission had been interrupted in the country for a period of at least 36 months. Since then, measles circulation in Spain consisted of sporadic imported cases or small outbreaks linked with them. However, Spain also experienced an increase in incidence before the COVID-19 pandemic despite persistently high vaccination coverage (more than 95% received 1 dose of MMR vaccine and 91.5% received 2 doses of MMR vaccines from the 2016 cohort; Instituto de Salud Carlos III (2022)). In 2019, 287 cases were reported with an incidence of 6,1 cases per million of inhabitants [Instituto de Salud Carlos III (2020)]. One objective of the strategic plan for the elimination of measles and rubella in Spain, updated in 2021 (2021–2025), is to strengthen the surveillance system and outbreak response (Consejo Interterritorial del Sistema Nacional de Salud, 2021). Indeed, this is essential to maintain the endemic measles elimination status and requires increased efforts in data collection and interpretation (World Health Organization, 2018).

In addition to epidemiological surveillance, the use of molecular tools provides insights into the circulation of measles viruses and helps understanding whether there is endemic transmission or multiple introductions of MeV in a certain region or country (Santibanez et al., 2017). Molecular surveillance of MeV worldwide is currently based on the determination of the genotype by sequencing 450 nucleotides (nt) of the C-terminus region of the nucleoprotein gene (N450; World Health Organization, 2018). In 2018, only four MeV genotypes were circulating worldwide according to WHO (B3, D4, D8, and H1) and in Europe the vast majority of reported sequences belonged to MeV B3 and D8 genotypes (Brown et al., 2019). WHO keeps a database of MeV sequences named Measles Nucleotide Surveillance (MeaNS). Within genotypes, each set of identical N450 sequences widely detected is designated as sequence variant or “named strain” by MeaNS (World Health Organization, 2015). The study of variants inside genotypes has allowed to facilitate molecular surveillance. However, the genetic variation of N450 sequences is limited and is insufficient in the elimination phase to distinguish chains of MeV transmission and to infer the origin of cases. As a matter of fact, most reported sequences in Spain from 2017 to 2020 belonged to the MVs/Dublin.IRL/8.16 (B3-Dublin) or the MVs/Gir Somnath.IND/42.16 (D8-Gir Somnath) variants [Instituto de Salud Carlos III (2020)]. Therefore, the collection of additional genomic information was proposed as a solution to increase the resolution in molecular surveillance.

Researches have focused on the MF-NCR which is the longest non-coding region of the MeV genome, GC-rich and highly variable (Penedos et al., 2015; Harvala et al., 2016; Gil et al., 2018; Bodewes et al., 2021). However, standard protocols are still not available for the analysis of MF-NCR sequences and for the criteria used to associate sequences in order to distinguish chains of transmission. Recently, a method based on a mathematical model was validated to support the interpretation of MF-NCR sequences in relation with epidemiology data (Penedos et al., 2022).

**Figure 1:**
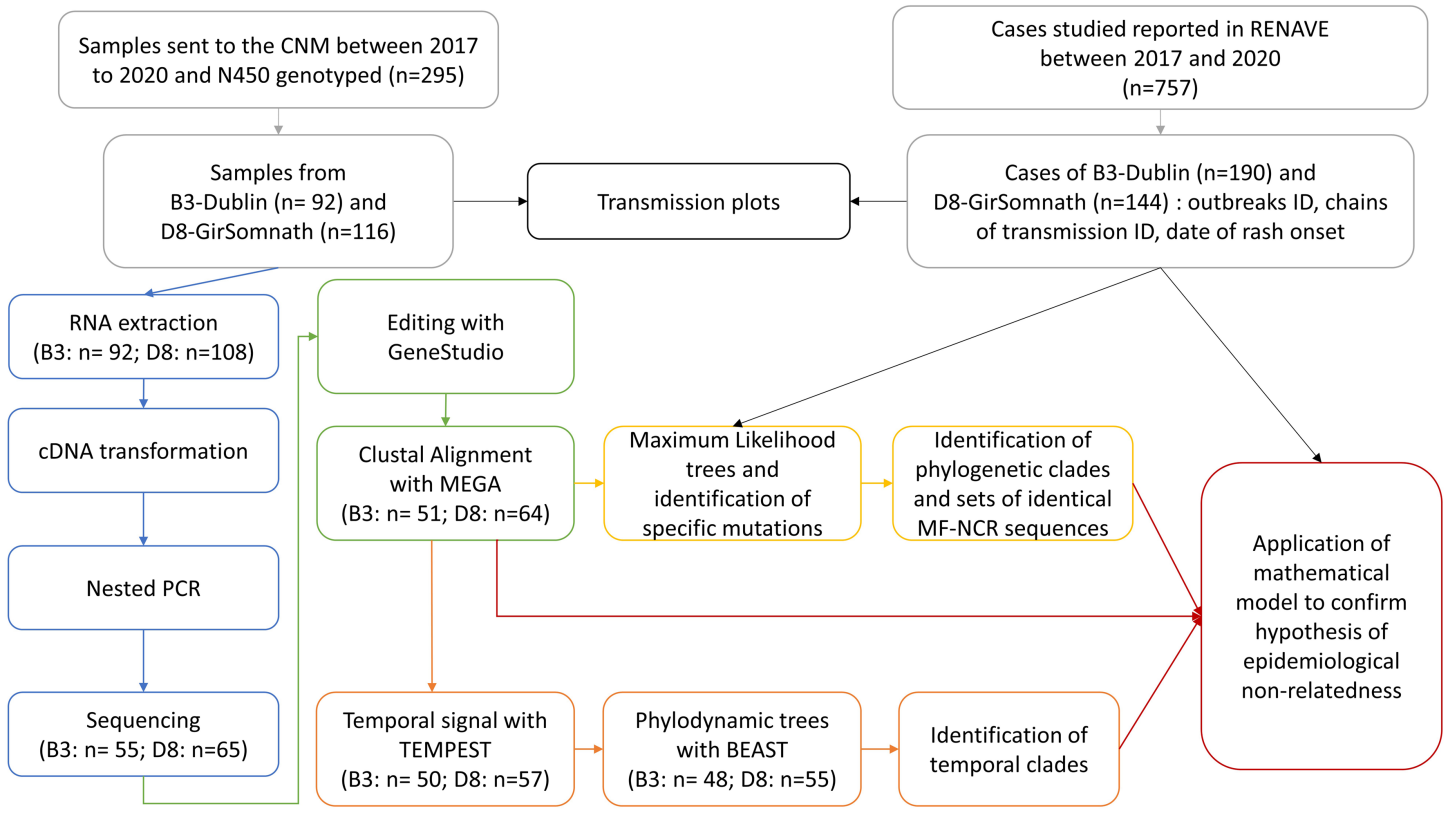
Scheme of the analysis pipeline consisting in phylogenetic and phylodynamic analyses coupled with a mathematical model.

The aim of the present study was to investigate whether the use of the MF-NCR region increases the resolution of measles surveillance by improving (i) discrimination between MeV with identical N450 sequences; and (ii) identification of chains of transmission and unknown importations. The pipeline of analyses consisted in phylogenetic and phylodynamic analyses coupled with a mathematical analysis (Figure 1). We evaluated its power to identify outbreaks and chains of transmission against two datasets containing (i) Spanish sequences available from the B3-Dublin and D8-Gir Somnath variants, and (ii) sequences from three well-described national outbreaks.

## Materials and methods

### Epidemiological data and analyses

In Spain, the regional public health services report measles cases to the National Epidemiological Surveillance Network (RENAVE) and complete a standardized case questionnaire *via* the Surveillance System electronic platform (SiViES in Spanish). The questionnaire includes items on demographic and clinical characteristics, complications, risk factors and laboratory results for diagnosis. For each confirmed case, we collected the following epidemiological information: date of rash onset, outbreak and chain of transmission identifier (as reported to WHO), contacts with other cases, importation status (case exposed outside of the country in the 7–21 days before symptom onset), type of exposure and molecular data including the WHO name and N450 variant or “named strain.” For our analyses of chains of transmission, we focused on three national outbreaks: (i) B3-Dublin in the region of Navarra in 2017; (ii) B3-Dublin in the region of Valencia in 2017–2018 (Pampaka et al., 2023); and (iii) D8-Gir Somnath in the regions of Madrid and Castilla-La-Mancha in 2019 (González-Praetorius et al., 2021). Table 1 presents the epidemiological characteristics of each of these outbreaks. Taken into account the high number of cases reported for these outbreaks, we also obtained transmission plots and considered the following parameters: (i) a median period from MeV exposure to rash onset of 14 days (min = 7 days, max = 21 days before onset), and (ii) an infectiousness period from 4 days before to 4 days after rash onset according to the RENAVE protocol [Instituto de Salud Carlos III (ISCIII), Red Nacional de Vigilancia Epidemiológica (RENAVE), 2016]. Figures were produced with R 4.1.2 (R Development Core Team, 2011). Networks were created using yED live^1^ using the available contact-tracing information.

**Table 1:** Characteristics of measles cases of three outbreaks in Spain.

Characteristics	2017 NAV 24	2017 VAL 2755	2019 MAD–CLM
First case	09/05/2017	01/12/2017	06/02/2019
Last case	26/06/2017	28/06/2018	25/08/2019
Total number of cases	28	154	99
Total number of chains of transmission	3 Laboral 2 (Work) Laboral 3 (Work) Hospital 1 (Nosocomial)	5 2793 (Nosocomial and Familial) 2846 (School) Carpool *UTE03-18 (Nosocomial)*	10 *670 (Imported)* 705 (Familial) 707 (Familial) 740 (Nosocomial) 783 (Familial) GU01 (Nosocomial and School and Familial) *2019GAL-01 (Nosocomial)*
Sex (%)			
Females	50.0	56.5	52.5
Males	50.0	43.5	47.5
Age group (%)			
< 1	0	7.2	17.2
1–4	0	3.3	11.1
5–9	0	4.0	0
10–19	3.6	4.7	4.0
20–29	22.1	18.9	18.2
≥ 30	74.3	61.9	49.5
Vaccination status (%)			
Yes (at least 1 dose)	53.6	20.2	30.3
No	39.3	66.2	59.6
Unknown	7.1	13.6	10.1
Importation	Unknown	Romania	Ukraine
Travel history (%)			
Yes	7.2	8.4	7.1
No	92.8	89.0	82.8
Unknown	0	2.6	10.1

Only laboratory-confirmed cases from B3-Dublin and D8-Gir Somnath variants were included in this table. We were not able to obtain sequences for the chains of transmission shown in italic.

### Clinical specimens

Between 2017 and 2020, 295 samples were received at the National Reference Laboratory for Measles and Rubella at the National Center of Microbiology (CNM) and genotyped according to WHO protocols as previously described (Mosquera et al., 2005). For our analysis, we considered 208 samples as they belonged to the Mvs/Dublin.IRL/8.16 (B3-Dublin; *n* = 92) or the MVs/Gir Somnath.IND/42.16 (D8-Gir Somnath; *n* = 116) variants. The majority of the selected samples were nasopharyngeal exudates (*n* = 154) and urines (*n* = 33).

### RNA extraction, retro-transcription, PCR and sequencing

RNA was extracted from 200 μl of sample using the Quick RNA Viral kit (Zymo), according to the manufacturer’s recommendations, to obtain 15 μl of final eluate. Synthesis of cDNA was performed according to the manufacturer’s protocol for SuperScript IV First-Strand Synthesis System Kit (ThermoFisher) using 5 μl of the eluate.

Amplification of the MF-NCR as a single fragment was performed using primers previously described (Gil et al., 2018) with the following modifications. The first amplification was carried out using MyTaq Reaction buffer (1 ×) including 3 mM MgCl_2_ and 1 mM dNTPs, 5 μl of cDNA as a template, 0.5 μM of each primer (MV_F1: 5′-CAAGATAGTAAGAATCCAGGCAG-3′ and MV_R1: 5′-ACT TTGTAGCTTGCACTTCC-3′) obtained from Sigma-Aldrich^©^, 2.5 U of MyTaq HS DNA Polymerase (Bioline) and 0.5 U of Pfu Turbo (Agilent Technologies), to a final volume of 50 μl. After a denaturation step of 2 min at 95°C, amplification was performed for 35 cycles at 95°C for 30 s, annealed at 55°C for 30 s and elongated at 72°C for 2 min, followed by a final elongation step at 72°C for 10 min. The second amplification was conducted similarly with 2 μl of PCR products as template and 0.5 μM of each primer (MV_F2: 5′-CGTGATCATAAATGATGACCAAGGAC-3′ and MV_R2: 5′-TTGTAGCTTGCACTTCCTAYYCC-3′). Amplicons were purified using Illustra ExoProStar 1-Step (GE Health Care Life Science) according to manufacturer’s instructions. Amplicons were sequenced with the ABI Big Dye Terminator Cycle Sequencing Kit (Applied Biosystems) for Sanger sequencing using the MV_F2 and MV_R2 primers described above as well as the following additional primers: MV_F4 (5′-AACTTAGGGCCAAGGAAYAYAC-3′) and a newly designed primer MV_R6 (5′-GGTGTGCCTRVVTGYG-3′). All primers were prepared to 5 μM in betaine 5 M and used in a 1:5 dilution.

### Sequence edition, phylogenetic analyses, and identification of sets of identical MF-NCR sequences

Sequences were assembled and edited using GeneStudio 2.2.0.0^2^ to ensure that the region of the MF-NCR was supported by the sequencing obtained with the two amplified DNA strands. Every consensus sequence was named in accordance with the WHO’s standard nomenclature (World Health Organization, 2012). Sequences were aligned using MEGA 11’s ClustalW alignment with default settings (Tamura et al., 2021). IQ-TREE v1.6.12 (Trifinopoulos et al., 2016) was used to generate a maximum likelihood (ML) tree using the best fitted nucleotide substitution model for each dataset and region previously identified using IQ-TREE’s model finder. A B3 sequence from the United Kingdom in 2013 (KT732215) and a D7 sequence from United States in 2003 (JN635410) obtained from GenBank were used as outgroups as previously described (Bodewes et al., 2021). The reliability of the phylogenetic analyses at each branch node was estimated by the UltraFast bootstrap method using 1,000 replicates (Minh et al., 2013). Phylogenetic trees were edited using MEGA (Tamura et al., 2021). After multiple sequence alignment, each set of identical MF-NCR sequences was identified and named with the earliest sequence name (according to the WHO convention; Gil et al., 2018). Each group of sequences that shared a unique common ancestor were referred to as a clade (Lemey et al., 2009).

### Phylodynamic analyses

Phylodynamic analyses were conducted as presented in a previous study (Penedos et al., 2022). To determine the time of divergence between samples, we estimated the time of emergence of the most recent common ancestor (MRCA) of a group of sequences using the Bayesian Markov Chain Monte Carlo (MCMC) coalescent method implemented in BEAST v1.10.4 (Bouckaert et al., 2019). First, phylogenetic trees, obtained as described above, and sample dates (defined as date of rash onset) were analyzed in TempEst v1.5.3 (Rambaut et al., 2016) using a regression of root-to-tip genetic distances against sampling time to verify that a temporal signal is present in the dataset and to remove outliers. Then, files were prepared in BEAUti v1.10.4. (Rambaut et al., 2016) and BEAST analysis was carried out using a strict molecular clock model and the general time reversible (GTR) substitution model with ten gamma heterogeneity categories. A coalescent Bayesian Skygrid plot population growth model was used to account for variations in population size. Finally, the results of 2 runs were processed into single log and tree files which were then used to create a maximum clade credibility (MCC) tree using LogCombiner v1.10.4 and TreeAnnotator v1.10.4 (both part of the BEAST package). Convergence was assessed using Tracer v1.7.1. The consensus BEAST-inferred phylogenetic trees produced were plotted using FigTree v1.4.3^3^.

### Model to exclude relatedness between sample pairs

For samples belonging to different phylogenetic clades inside the same outbreak, we applied a mathematical model to exclude epidemiological relatedness as previously described (Penedos et al., 2022). We gave the model three inputs: (i) the onset dates for the two samples for which the epidemiological relatedness is being tested; (ii) the time of the earliest known case of the outbreak based on epidemiological data; and (iii) the number of substitutions between the two samples. The number of observed substitutions between two samples was calculated from the number of characters that differed between the sequences in a multiple sequence alignment. We then used the tool published by Penedos et al. (2022) to calculate Poisson probabilities and maximum expected substitutions. We used a substitution rate of 1.94 × 10^3 for B3 sequences and 2.39 × 10^3 for D8 sequences and a Poisson interval of 0.95 (Penedos et al., 2022). We considered that a sample pair was unrelated when it had higher number of substitutions than the expected maximum substitution number as calculated by the model for this time frame.

## Results

Out of the 208 samples considered for our analyses, we obtained the MF-NCR sequence of 55 (60%) and 65 (56%) cases for B3-Dublin and D8-GirSomnath, respectively. Those results are comparable to what was observed previously with a OneStep RT-PCR (Penedos et al., 2015, 2022) but cDNA synthesis insure longer stability of the samples.

### Confirmation of outbreaks based on the analysis of the MF-NCR

**Figure 2:**
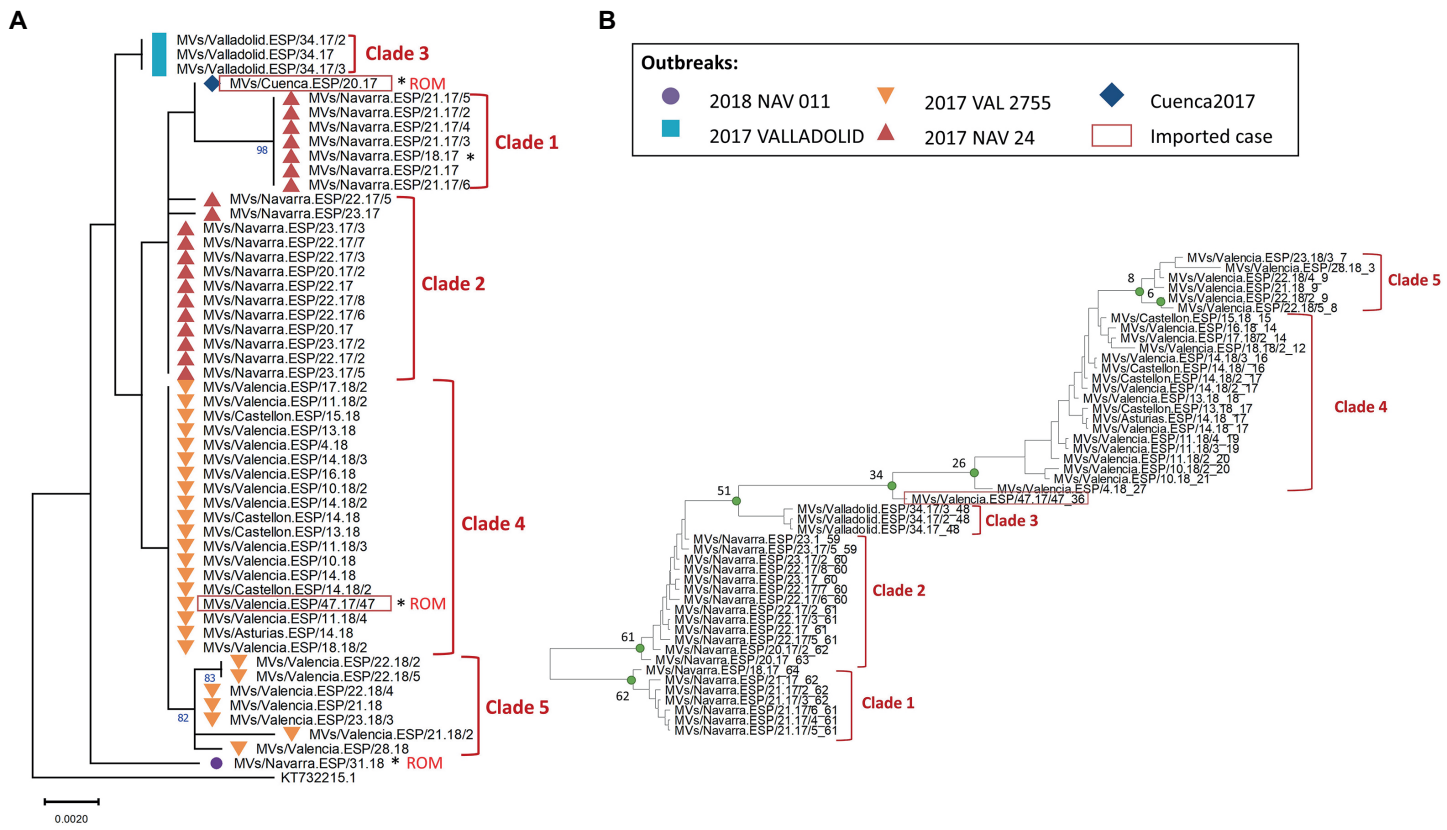
**(A)** Maximum likelihood tree of MeV genotype B3 strains based on the MF-NCR region using the HKY + F substitution model. Values on branches are shown as percentages based on 1,000 bootstrap replicates, only values above 80% are presented. The colored symbols represent the different outbreaks as described in the legend (NAV: Navarra, VAL: Valencia) with the codes used in SiViEs and in WHO reports. Imported cases are shown in red squares with their origin (ROM: Romania). Index cases are shown with an asterisk (^*^). The tree was rooted with respect to a B3 reference strain from United Kingdom in 2013 and is labeled with the accession number of GenBank. Sequences are named according to WHO convention. **(B)** BEAST maximum credibility time-scaled phylogenetic tree for MeV genotype B3 samples based on the MF-NCR region. The number of weeks since the date of onset of the last case is added to the WHO name after the underscore. Edge support values are given by the BEAST tree posterior values, well supported edges (PP > 0.9) are represented by green circles. The estimated week before the date of onset of the last case of the MRCA is shown at the corresponding node for each clade. Imported cases are shown in red squares. The numbered clades are discussed in the manuscript’s text.

For samples belonging to the B3-Dublin variant, clean MF-NCR sequence data were obtained for 51 cases (four were excluded for poor sequence quality). Sequences were obtained from five outbreaks detected between 2017 and 2018. The three largest outbreaks were found in the regions (autonomous communities): Castilla y León (2017 VALLADOLID), Navarra (2017 NAV 24) and Valencia (2017 VAL 2755). The phylogenetic analyses and the identification of specific mutations resulted in the detection of 12 different sets of identical MF-NCR sequences (Supplementary Figure S1), most of them grouped into five phylogenetic clades (Figure 2A). While the outbreak of Valladolid (Castilla y León region) corresponded to a single set of identical MF-NCR sequences, four sets of identical MF-NCR sequences, grouped into two clades, were detected inside the outbreak of Navarra region and five sets of identical MF-NCR sequences grouped into two clades were found for the outbreak in the Valencia region. Two sequences (MVs/Cuenca.ESP/20.17 and MVs/Navarra.ESP/31.18) had a unique set of substitutions in our dataset and we could not sequence additional samples for the associated outbreaks. The analysis of the available epidemiological information also showed that the index cases of the outbreaks of Cuenca (Castilla-La Mancha region), Navarra (2018 NAV 011) and the Valencia region (2017 VAL 2755) were related to importations from Romania. The outbreak in Valladolid was also related to an importation from Romania but we could not sequence the index case.

To confirm our results, we conducted a phylodynamic analysis on the entire dataset after removing the sequence of MVs/Cuenca.ESP/20.17 as the two cases of this outbreak were related to importation. Our temporal analyses suggested that two sequences were temporal outliers (MVs/Valencia.ESP/21.18/2 and MVs/Navarra.ESP/31.18). After removing these temporal outliers, we conducted a phylodynamic analyses of the remaining 48 sequences. The results reinforced the phylogeny and supported the existence of five phylodynamic clades (defined by a node supported by posterior probability value (PP) above 0.95; Figure 2B). The estimated time of emergence of each clade was consistent with the date of rash onset of the index cases.

**Figure 3:**
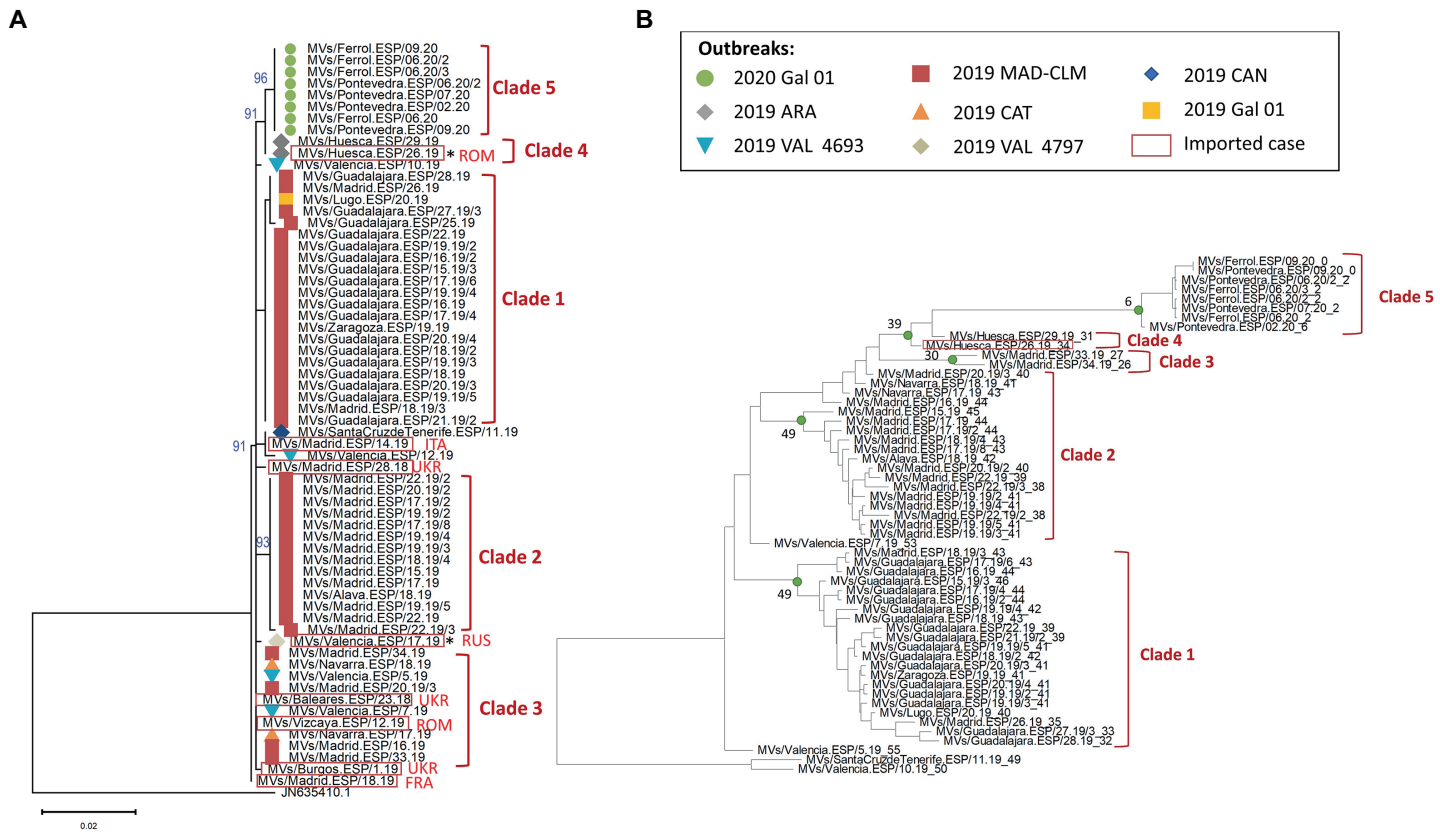
**(A)** Maximum likelihood tree of MeV genotype D8 strains based on the MF-NCR region using the TN + F substitution model. The colored symbols represent the different outbreak as described in the legend (Gal: Galicia, MAD: Madrid, CLM: Castilla-La Mancha, ARA: Aragón, CAT: Cataluña, CAN: Canarias, VAL: Valencia) with the codes used in SiViEs and in WHO reports. Imported cases are shown in red squares with their origin (ROM: Romania, ITA: Italia, UKR: Ukraine, RUS: Russia, FRA: France). Index cases are shown with an asterisk (^*^). The tree was rooted on a D7 reference strain from India in 2003 and is labeled with the accession number of GenBank. **(B)** BEAST maximum credibility time-scaled phylogenetic tree for MeV genotype D8 samples based on the MF-NCR region as presented for B3. The estimated week before the date of onset of the last case of the MRCA is shown at the corresponding node for each clade.

We apply the same methodology for MeV belonging to the D8-Gir Somnath variant. MF-NCR sequences were obtained for 64 samples (one was excluded for poor sequence quality) which correspond to eight outbreaks reported between 2018 and 2020 in Spain. We identified 15 sets of identical MF-NCR sequences, most of them grouped into five phylogenetic clades (Figure 3A; Supplementary Figure S2). Six sets of identical MF-NCR sequences, mostly grouped into three clades, were detected in the largest D8 outbreak from 2019 in the regions of Madrid and Castilla-La Mancha (2019 MAD–CLM) which was likely associated with an importation from Ukraine (the sequence of this imported case was not available). Our analyses confirmed the data from contact tracing, conducted by the public health authorities from regions, which showed that the case from Lugo (province from the region of Galicia) was part to the 2019 MAD–CLM outbreak, as it belongs to the same set of identical MF-NCR sequences than the cases of Guadalajara (Supplementary Figure S2). Eight imported cases of various origin were sequenced, two of them were the index cases of the outbreaks in the regions of Aragón (2019 ARA) and Valencia (2019 VAL 4797), and had a unique set of substitution in our dataset (Supplementary Figure S2). We could not obtain MF-NCR sequences for more cases associated with these outbreaks. In addition, the cases belonging to the outbreak of Galicia in 2020 formed a well-supported phylogenetic clade and were associated with an importation from Romania, but we could not sequence the index case. The case from Santa Cruz de Tenerife (Canary Islands) was epidemiologically linked to an imported case from Italy. A case from Madrid, that belonged to the same set of identical MF-NCR sequences, was also associated with importation from Italy. We also found that the sequences from the outbreak in the region of Valencia in 2019 (2019 VAL-4693) were not grouped into a single phylogenetic clade and two of them had an identical sequence to the ones from the outbreaks of Madrid/Castilla-La Mancha (2019 MAD–CLM) and Cataluña (2019 CAT; chain of transmission in Navarra).

Our temporal analyses showed two outliers (MVs/Guadalajara.ESP/25.19 and MVs/Valencia.ESP/12.19). After removing temporal outliers, six imported sporadic cases and the imported index case of the outbreak 2019 VAL 4797, as MF-NCR sequences could not be obtained for the two secondary cases, we performed the phylodynamic analysis on 55 sequences. The results supported the existence of five clades (posterior probability > 0.95), including two inside the 2019 MAD–CLM outbreak (Figure 3B). The clade 3, based on phylogeny, was not supported by the phylodynamic analysis and only two cases (MVs/Madrid.ESP/33.19 and MVs/Madrid.ESP/34.19) formed a well-supported phylodynamic clade (Clade 3).

### Deciphering chains of transmission inside the outbreaks

We then explored if (i) the MF-NCR provided sufficient resolution to identify chains of transmission inside the outbreaks; and (ii) the phylogenetic clades inside the outbreaks derived from each other by accumulation of substitutions or if there were the results of two independent importations.

**Figure 4:**
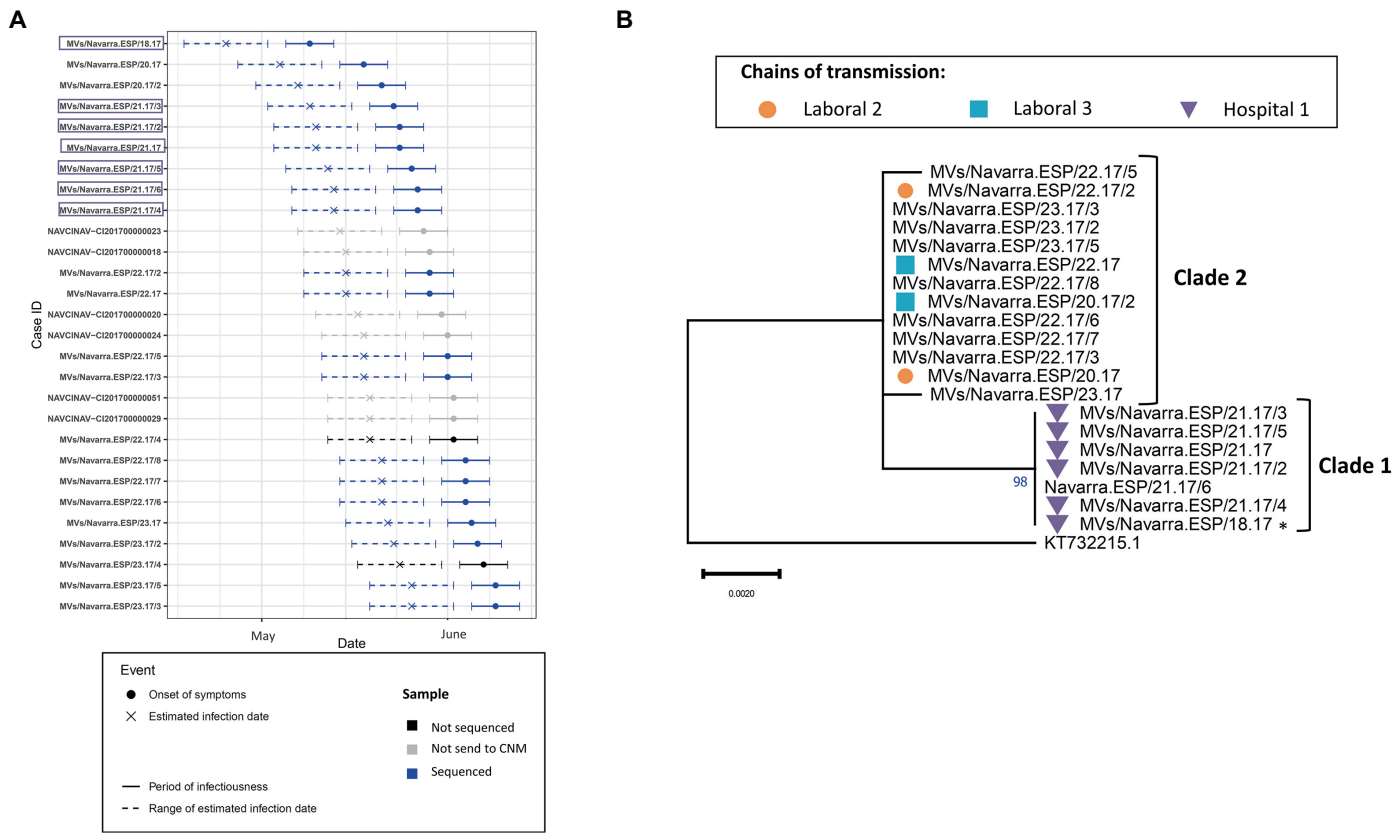
**(A)** Transmission plot for the outbreak of Navarra in 2017 showing the onset of symptoms, the estimated infection date, the period of infectiousness and the range of estimated infectious date as described in Material and Methods. Samples that were not send to the CNM are shown in gray, those that were sent but not sequenced for the MF-NCR (for technical reasons or because they were from other variants) are in black and the ones that were sequenced for the MF-NCR are in blue. Purple squares show the samples belonging to the clade 1. **(B)** Maximum likelihood tree of the strains belonging to the Navarra outbreak of 2017 based on the MF-NCR region using the HKY + F substitution model. The colored symbols represent the different chains of transmission as defined by contact-tracing with the codes used in SiViEs and in WHO reports. Index case is shown with an asterisk (^*^). The clades identified with the phylodynamic analysis are indicated in bold.

The outbreak of Navarra in 2017 (2017 NAV 24) contained 28 laboratory-confirmed cases from which we obtained 20 MF-NCR sequences. First, we confirmed that MeV belonging to both phylogenetic clades were circulating simultaneously in the region in May and June 2017 (Figure 4A). Then, using epidemiological data available for the chains of transmission, we found that Clade 1 was associated with nosocomial transmission in a hospital whereas Clade 2 was associated with two chains of transmission in working places (Figure 4B). We were not able to identify a case that would link the two clades and the probabilistic model predicted that MF-NCR sequences should only differ by up to one substitution in 3–5 weeks interval since the first case of the outbreak. We found that Clade 1 differed from Clade 2 by four substitutions (A4344G, A4405G, A4406G, and A4407G; Supplementary Figure S1) and therefore it is unlikely that the two clades are related in the time frame being considered. This is in agreement with the time-scaled phylogeny obtained with BEAST (Figure 2B), which estimates that the MRCA for these clades occurred 9 weeks prior to clade 1, when there was no measles circulating in the region. The index case of Clade 1 had been visited by relatives from Portugal, however the index case of Clade 2 is unknown.

**Figure 5:**
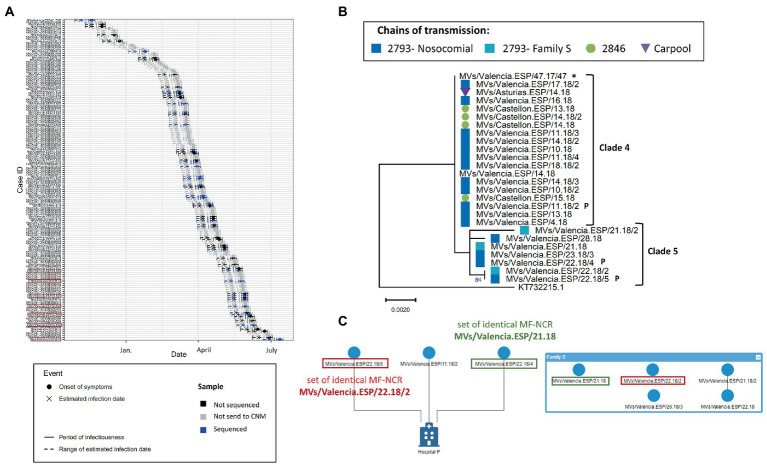
**(A)** Transmission plot for the outbreak of Valencia in 2017–2018. Red squares show the samples belonging to the clade 5. **(B)** Maximum likelihood tree of the strains belonging to the Valencia outbreak of 2017–2018 on MF-NCR region using the HKY + F substitution model. The colored symbols represent the different chains of transmission as defined by contact-tracing with the codes used in SiViEs and in WHO reports. Index case is shown with an asterisk (^*^). The clades identified with the phylodynamic analysis are indicated in bold. Sequences marked with a P belong to the cases related with the hospital P. **(C)** Network of transmission for the hospital P and the family S. Squares of color show the different sets of identical MF-NCR sequences shared by the family S and hospital P.

Following the same logic for the outbreak of Valencia in 2017–2018 (2017 VAL 2755), we analyzed the 27 MF-NCR sequences that were representative of the 148 laboratory-confirmed cases as they uniformly covered the duration of the outbreak (Figure 5A). We observed that the cases of Clade 5 had later dates of onset in the outbreak compared to cases in Clade 4 and could have been infected by earlier cases including those for which we did not obtain a MF-NCR sequence (Figure 5A). Clade 4 consisted of cases with identical sequences from three chains of transmission in two provinces of the region of Valencia (Valencia and Castellón), and in the region of Asturias. Epidemiological information indicates that the cases from Castellón and Asturias were infected in Valencia, supporting the results of the phylogenetic analyses. The cases in Clade 5 belonged to two chains of transmission: one related to nosocomial infection, which started with cases from Clade 4, and another related to intra-familial (Family S) transmission (Figure 5B). Clade 5 share a common substitution (C5027T; Supplementary Figure S1) with Clade 4. The network analysis based on contact tracing information showed a link between one case in Clade 4 and two cases in Clade 5 as they all were exposed in the same hospital P (Figure 5C). Within Clade 5, an epidemiological link could not be identified between the cases in the hospital and the cases in the family S but the phylogenetic analysis showed two sets of identical MF-NCR sequences (MVs/Valencia.ESP/21.18 and MVs/Valencia.ESP/22.18/2) that included cases from both the family S and the hospital P. According to the mathematical model the maximum number of expected substitutions since the index case was between four and five. We observed a number of substitutions between one and four and therefore could not exclude that the cases of Clade 4 and Clade 5 were related.

**Figure 6:**
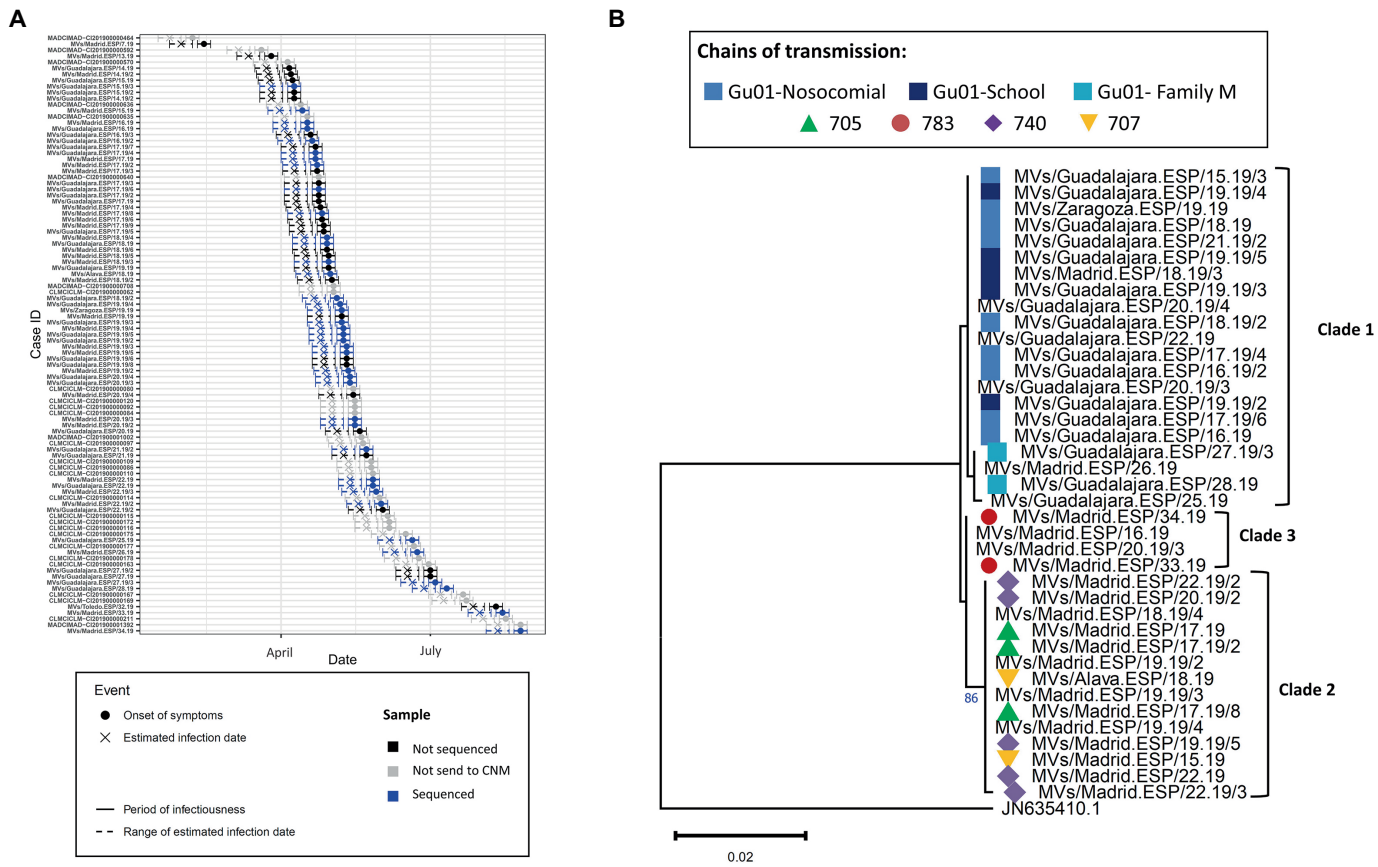
**(A)** Transmission plot for the outbreak of Madrid/Castilla-La Mancha 2019. **(B)** Maximum likelihood tree of the strains belonging to the outbreak based on the MF-NCR region using the HKY + F substitution model. The colored symbols represent the different chains of transmission as defined by contact-tracing with the codes used in SiViEs and in WHO reports. The clades identified with the phylodynamic analysis are indicated in bold.

Finally, we analyzed the outbreak of the regions Madrid and Castilla-La Mancha of 2019 (2019 MAD–CLM). According to the onset of rash, cases from clades 1 and 2 were circulating simultaneously from January to September 2019 (Figure 6A). Sequences from clade 1 were associated with three chains of transmission, one related to nosocomial transmission, one associated with transmission in a day care and one intra-familial. While most of the cases were reported in Guadalajara, two cases were from Madrid and one case from Zaragoza (region of Aragón; Figure 6B). Clade 2 was associated with three chains of transmission in Madrid and Álava (País Vasco), one was associated with nosocomial transmission (740), which showed identical sequence except for one case (Supplementary Figure S2). Clade 3 was associated with a single chain of transmission, but two earlier cases showed identical sequences. We did not identify an epidemiological link between the three clades. The model predicted a maximum of three substitutions between samples from Clades 1 and 2. However, we observed five substitutions suggesting the cases from the two clades were not related. While the index case of Clade 2 was associated with importation from Ukraine, we were not able to identify the index case of Clade 1.

## Discussion

Since the declaration of elimination of endemic measles in Spain by the WHO in 2017, recurrent outbreaks have been sporadically reported and caused mostly by two N450 variants: B3-Dublin and D8-Gir-Somnath. In the present study, the analysis of the MF-NCR region was evaluated as a method to increase discrimination between viruses with identical N450 sequences in order to improve the ability to infer chains of transmission and outbreaks. Using cases from multiple outbreaks, our results suggest that the MF-NCR analysis could support the outbreaks identified by contact-tracing and traditional epidemiological studies. Moreover, the probabilistic method may be useful to decipher the relatedness of samples inside outbreaks, including the identification of chains of transmission and multiple viral introductions. For example, the outbreaks of Navarra in 2017 and Madrid/Castilla-La Mancha in 2019 were probably the result of two independent introductions that were not identified by the study of N450 variants in combination with the epidemiological data. This result has two implications: (i) it shows the relevance of the method as a tool to direct contact tracing in order to identify index cases in context of simultaneous importations in the same region; (ii) it supports that endemic transmission was absent in Spain, given that the size of the importation-related outbreaks was smaller than previously found. The outbreak of Madrid/Castilla-La Mancha highlights a situation where epidemiological methods are challenged, and N450 sequencing is not sufficiently discriminative to complement them. Several contacts were established between Madrid and Guadalajara due to their geographical proximity, making the accurate attribution of individual cases to a particular outbreak impossible when based only on time and place. That is where the analyses MF-NCR showed the most added-value, as we were able to suggest two independent introductions from which one was mostly related to Guadalajara but with some cases from Madrid.

The use of the entire MF-NCR region is more cost-effective and less labor-intensive, while bringing similar resolution, than other methods such as the use of two or three different regions of the genome (Bodewes et al., 2021; Kim et al., 2021) or whole genome sequencing (WGS; Penedos et al., 2015). In addition, our protocol allows to increase stability of the samples together with high yield of MF-NCR amplifications compared to what was observed with described protocol (Penedos et al., 2022). Phylodynamic analyses showed to be a powerful tool to confirm the phylogenetic analyses, to characterize outbreaks and to support the interpretation of contact information. Nevertheless, our study shows that the relatedness model represents a good alternative, as it is faster and does not require bioinformatics analyses. Rather than assessing relatedness inside a chain of transmission, the model should be used to unlink cases when multiple introductions are suspected.

One limitation of our study is associated with the limited sampling rate as we obtained MF-NCR sequences for as little as 18% of the cases of the Valencia region outbreak. However, the sequences obtained provided a good coverage of the outbreak both temporally and geographically (Figure 5A). In addition, in large outbreaks more than one N450 sets of identical MF-NCR sequences can be detected because of a substitution in the N450 region, which could occur independently of those in the MF-NCR region. We focused on the analysis of the MF-NCR sequences inside the predominant variant of the outbreak, however, future studies should include sequences from other N450 sets of identical MF-NCR sequences belonging to same outbreak to evaluate the pertinence of the MF-NCR region in those situations. Phylogeny and phylodynamic analyses could also be conducted with concatenated N450 and MF-NCR sequences to reflect changes in both genomic regions. Finally, the MF-NCR region alone is potentially not sufficient to identify all chains of transmission, even though as it has been shown to provide comparable phylogenetic resolution to that of WGS (Penedos et al., 2015). Several explanations can be proposed for samples belonging to different epidemiological chains of transmission with an identical MF-NCR sequence: (i) either cases have the same infectious origin, (ii) two separate chains of transmission may have acquired the same mutations, or (iii) the genetic diversity in circulating MeV may be insufficient, in which case WGS may help distinguishing them.

The number of measles patients surged worldwide in 2019 before the COVID-19 pandemic and the consequences that the recent conflict in Ukraine could have on the circulation of the virus in Europe are still unknown. However, the previous conflict of the Donbas region caused a sharp decline on vaccine coverage in Ukraine and a large outbreak that extended to different European countries (Lancet, 2018). In addition, with the decline in the vaccination coverage worldwide, there is an increased risk to observe sporadic outbreaks linked with importations (Durrheim et al., 2014; Lee et al., 2019). In this context, it is of primary importance to find new methods to support epidemiological evidence and increase the resolution of molecular surveillance for countries in the post-elimination phase. We showed here the added value of the MF-NCR analysis to reveal unknown importations. However, this method must be considered as a complement of the variant analysis based on recommended genotyping methods, not in place of it. It might also be a useful tool in cases where epidemiological evidence is not available, including to identify the origin of a imported cases by comparing them with reference sequences from different countries as already suggested (Kim et al., 2021). To improve this comparison, international efforts must continue for the production and sharing of genomic information on measles outbreaks, including MF-NCR sequences.

In conclusion, the multi-faceted approach described here is relevant to track importations and verify the maintenance of measles elimination status. The analysis of the MF-NCR could be added to N450 sequencing in the protocol for routine molecular surveillance to support epidemiological studies in elimination settings. In addition of phylogenetic analyses, we also propose the use of a previously described mathematical model (Penedos et al., 2022) to establish clear criteria for assessing the epidemiological relatedness between identified MeV cases.

## Data availability statement

All the MF-NCR sequences (including the stop codon of the M gene and the start codon of the F gene, i.e., 1018 nt) included in this study were deposited in ON755137-ON755141, ON755143-ON755156, ON792426-ON792428, ON792430-ON792434 and ON792437-ON792520 and ON792522-ON792525.

## Ethics statement

The samples used in this work were received by the National Reference Laboratory for Measles and Rubella at the CNM, in the context of the National Measles and Rubella Elimination Plan and used in accordance with the requirements of Spanish biomedical research law (Ley 14/2007 de Investigación Biomédica). The protocol was approved by the Comité de Ética de la Investigación of the Instituto de Salud Carlos III (approval no. CEI PI 35–2015). Written informed consent from the participants was not required to participate in this study in accordance with the national legislation and the institutional requirements.

## MMR study group

**Aragón.**

Ana Martínez Sapiña, Servicio de Microbiología, Hospital Universitario Miguel Servet, Zaragoza, Spain.

Ana Cebollada, Dirección General de Salud Pública de Aragón, Zaragoza, Spain.

**Castilla La Mancha.**

Alejandro González-Praetorius, Sección de Microbiología, Hospital Universitario de Guadalajara, Guadalajara, Spain.

María Victoria García-Rivera, Servicio de Epidemiología, Dirección General de Salud Pública de Castilla-La Mancha, Toledo, Spain.

**Castilla y León.**

Silvia Rojo-Rello, José Mª Eiros-Bouza and Raúl Ortiz-de-Lejarazu, Servicio de Microbiología del Hospital Clínico Universitario de Valladolid, Valladolid, Spain.

Cristina Ruiz-Sopeña and Mª Jesús Rodríguez Recio, Servicio de Epidemiología. Dirección General de Salud Pública de Castilla y León. Valladolid, Spain.

**Comunidad Foral de Navarra.**

Ana Navascués, Servicio de Microbiología Clínica, Hospital Universitario de Navarra, Pamplona, Spain.

Manuel García-Cenoz, Instituto de Salud Pública y Laboral de Navarra, Pamplona, Spain.

**Comunidad de Madrid.**

Juan Carlos Sanz and Marta Pérez-Abeledo, Laboratorio Regional de Salud Pública de la Comunidad de Madrid. Dirección General de Salud Pública de la Comunidad de Madrid, Madrid, Spain.

Luis García-Comas, Inma Rodero-Garduño and Alba Nieto Juliá, Área de Vigilancia y Control de Enfermedades Transmisibles, Dirección General de Salud Pública de la Comunidad de Madrid, Madrid, Spain.

**Comunidad Valenciana.**

Beatriz Acosta-Boga, Servicio de Microbiología, Hospital Universitari i Politècnic La Fe, Valencia, Spain.

María Gil, Servicio de Microbiología, Hospital General de Castellón, Castellón, Spain.

Javier Roig, Katja Villatoro, Maite Castellanos and Isabel Huertas, Servicio de Vigilancia y Control Epidemiológico de la Comunidad Valenciana, Valencia, Spain.

Juan Bellido, Centro de Salud Pública de Castellón, Castellón, Spain.

María Sanz, Centro de Salud Pública de Manises, Manises, Spain.

**Galicia.**

Patricia Ordoñez-Barrosa, Servicio de Microbiología, Complejo Hospitalario Universitario de Ferrol, Ferrol, Spain.

Matilde Trigo-Daporta, Servicio de Microbiología, Complejo Hospitalario Universitario de Pontevedra, Pontevedra, Spain.

Isabel Losada-Castillo, Servizo de Calidade Asistencial, Dirección Xeral de Asistencia Sanitaria de Galicia, Santiago de Compostela, Spain.

Alberto Malvar Pintos, Servizo de Epidemioloxia, Dirección Xeral de Saúde Pública, Santiago de Compostela, Spain.

## Author contributions

CJ: technical work, data analysis and writing as main author. AG: technical work and data analysis. JM-C and NL-P designed the study, reviewed and assisted in the editing of the final version of the manuscript. JE, design for the study and revised the manuscript. AP assisted in the use of mathematical method and editing of the final version of the manuscript. AF-G designed the study, revised the manuscript and writing as main author. All authors contributed to the article and approved the submitted version.

## Funding

This work was supported by the “Instituto de Salud Carlos III” (PI15CIII/00023, PI19ICIII/0041). AG was funded by CIBER de Epidemiología y Salud Pública (CIBERESP), ISCIII. CJ was funded by the ECDC/EUPHEM fellowship.

## Conflict of interest

The authors declare that the research was conducted in the absence of any commercial or financial relationships that could be construed as a potential conflict of interest.

## Publisher’s note

All claims expressed in this article are solely those of the authors and do not necessarily represent those of their affiliated organizations, or those of the publisher, the editors and the reviewers. Any product that may be evaluated in this article, or claim that may be made by its manufacturer, is not guaranteed or endorsed by the publisher.

## Footnotes

^1^ https://www.yworks.com/yed-live/

^2^ https://sourceforge.net/projects/genestudio/

^3^ http://tree.bio.ed.ac.uk/software/figtree/

## Supplementary material

Supplementary Figure S1 Alignment of the MF-NCR region for the 12 sets of identical MF-NCR sequences found in B3. Regions without substitutions are not shown in the sake of place.

Supplementary Figure S2 Alignment of the MF-NCR region for the 15 sets of identical MF-NCR sequences found in D8. Regions without substitutions are not shown in the sake of place.
