# Supplementary material for: Utility of MF-non coding region for measles molecular surveillance during post-elimination phase, Spain, 2017–2020
Source: Front Microbiol. 2023 May 22;14:1143933. doi: 10.3389/fmicb.2023.1143933 (PMC10240958; doi:10.3389/fmicb.2023.1143933)
Supplement: Supplementary Figure S1 — Alignment of the MF-NCR region for the 12 sets of identical MF-NCR sequences found in B3. Regions without substitutions are not shown in the sake of place. [file Data_Sheet_1.zip › Figure S1.pdf]

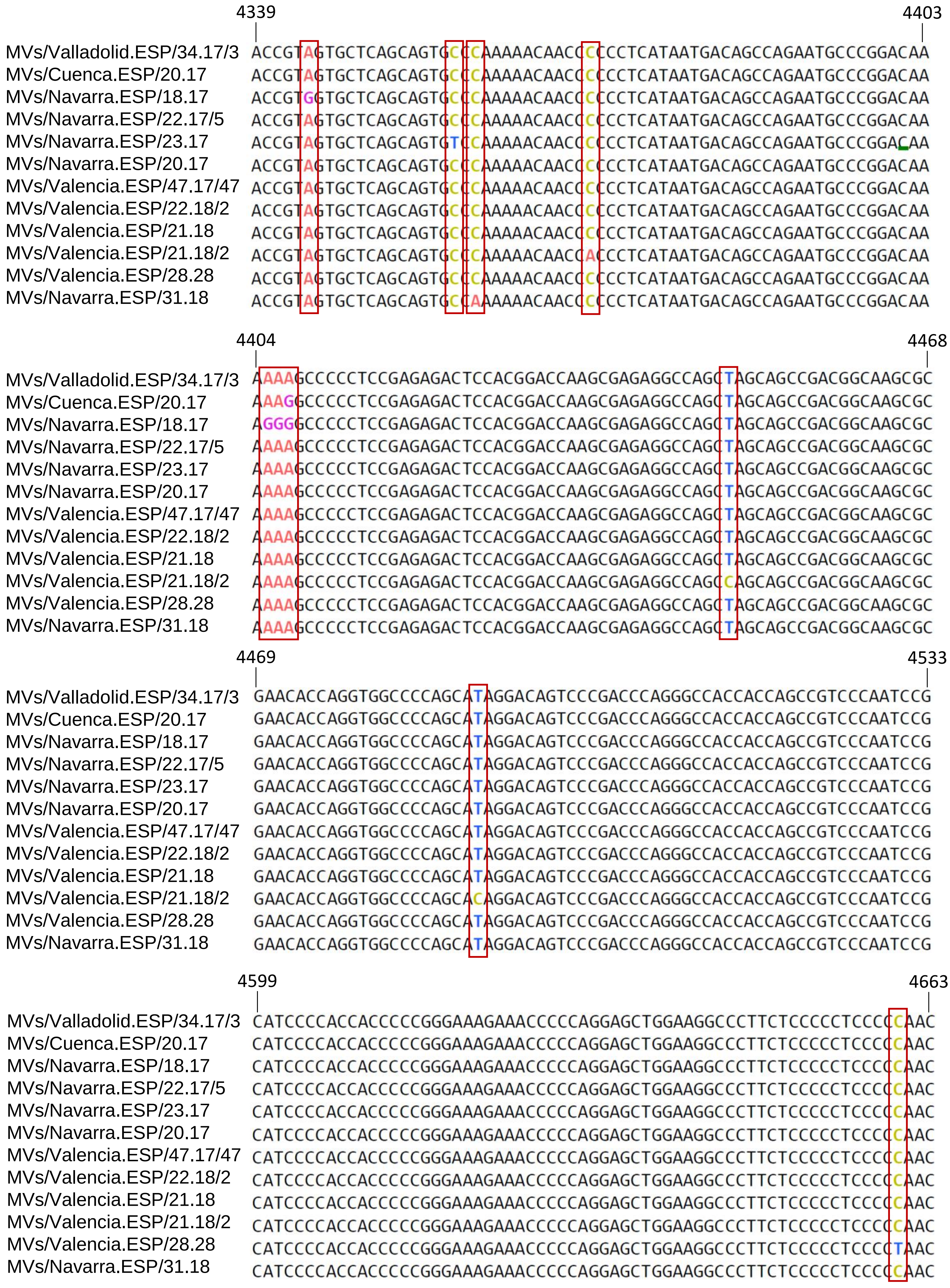
4339
4403
MVs/Valladolid.ESP/34.17/3 ACCGTAGTGCTCAGCAGTGCCCAAAAACAACCCCCCTCATAATGACAGCCAGAATGCCCGGACAA
MVs/Cuenca.ESP/20.17 ACCGTAGTGCTCAGCAGTGCCCAAAAACAACCCCCCTCATAATGACAGCCAGAATGCCCGGACAA
MVs/Navarra.ESP/18.17 ACCGTGGTGCTCAGCAGTGCCCAAAAACAACCCCCCTCATAATGACAGCCAGAATGCCCGGACAA
MVs/Navarra.ESP/22.17/5 ACCGTAGTGCTCAGCAGTGCCCAAAAACAACCCCCCTCATAATGACAGCCAGAATGCCCGGACAA
MVs/Navarra.ESP/23.17 ACCGTAGTGCTCAGCAGTGTCCAAAAACAACCCCCCTCATAATGACAGCCAGAATGCCCGGACAA
MVs/Navarra.ESP/20.17 ACCGTAGTGCTCAGCAGTGCCCAAAAACAACCCCCCTCATAATGACAGCCAGAATGCCCGGACAA
MVs/Valencia.ESP/47.17/47 ACCGTAGTGCTCAGCAGTGCCCAAAAACAACCCCCCTCATAATGACAGCCAGAATGCCCGGACAA
MVs/Valencia.ESP/22.18/2 ACCGTAGTGCTCAGCAGTGCCCAAAAACAACCCCCCTCATAATGACAGCCAGAATGCCCGGACAA
MVs/Valencia.ESP/21.18 ACCGTAGTGCTCAGCAGTGCCCAAAAACAACCCCCCTCATAATGACAGCCAGAATGCCCGGACAA
MVs/Valencia.ESP/21.18/2 ACCGTAGTGCTCAGCAGTGCCCAAAAACAACCACCCTCATAATGACAGCCAGAATGCCCGGACAA
MVs/Valencia.ESP/28.28 ACCGTAGTGCTCAGCAGTGCCCAAAAACAACCCCCCTCATAATGACAGCCAGAATGCCCGGACAA
MVs/Navarra.ESP/31.18 ACCGTAGTGCTCAGCAGTGCCAAAAAACAACCCCCCTCATAATGACAGCCAGAATGCCCGGACAA
4404
4468
MVs/Valladolid.ESP/34.17/3 AAAAGCCCCCTCCGAGAGACTCCACGGACCAAGCGAGAGGCCAGCTAGCAGCCGACGGCAAGCGC
MVs/Cuenca.ESP/20.17 AAAGGCCCCCTCCGAGAGACTCCACGGACCAAGCGAGAGGCCAGCTAGCAGCCGACGGCAAGCGC
MVs/Navarra.ESP/18.17 AGGGGCCCCCTCCGAGAGACTCCACGGACCAAGCGAGAGGCCAGCTAGCAGCCGACGGCAAGCGC
MVs/Navarra.ESP/22.17/5 AAAAGCCCCCTCCGAGAGACTCCACGGACCAAGCGAGAGGCCAGCTAGCAGCCGACGGCAAGCGC
MVs/Navarra.ESP/23.17 AAAAGCCCCCTCCGAGAGACTCCACGGACCAAGCGAGAGGCCAGCTAGCAGCCGACGGCAAGCGC
MVs/Navarra.ESP/20.17 AAAAGCCCCCTCCGAGAGACTCCACGGACCAAGCGAGAGGCCAGCTAGCAGCCGACGGCAAGCGC
MVs/Valencia.ESP/47.17/47 AAAAGCCCCCTCCGAGAGACTCCACGGACCAAGCGAGAGGCCAGCTAGCAGCCGACGGCAAGCGC
MVs/Valencia.ESP/22.18/2 AAAAGCCCCCTCCGAGAGACTCCACGGACCAAGCGAGAGGCCAGCTAGCAGCCGACGGCAAGCGC
MVs/Valencia.ESP/21.18 AAAAGCCCCCTCCGAGAGACTCCACGGACCAAGCGAGAGGCCAGCTAGCAGCCGACGGCAAGCGC
MVs/Valencia.ESP/21.18/2 AAAAGCCCCCTCCGAGAGACTCCACGGACCAAGCGAGAGGCCAGCCAGCAGCCGACGGCAAGCGC
MVs/Valencia.ESP/28.28 AAAAGCCCCCTCCGAGAGACTCCACGGACCAAGCGAGAGGCCAGCTAGCAGCCGACGGCAAGCGC
MVs/Navarra.ESP/31.18 AAAAGCCCCCTCCGAGAGACTCCACGGACCAAGCGAGAGGCCAGCTAGCAGCCGACGGCAAGCGC
4469
4533
MVs/Valladolid.ESP/34.17/3 GAACACCAGGTGGCCCCAGCATAGGACAGTCCCGACCCAGGGCCACCACCAGCCGTCCCAATCCG
MVs/Cuenca.ESP/20.17 GAACACCAGGTGGCCCCAGCATAGGACAGTCCCGACCCAGGGCCACCACCAGCCGTCCCAATCCG
MVs/Navarra.ESP/18.17 GAACACCAGGTGGCCCCAGCATAGGACAGTCCCGACCCAGGGCCACCACCAGCCGTCCCAATCCG
MVs/Navarra.ESP/22.17/5 GAACACCAGGTGGCCCCAGCATAGGACAGTCCCGACCCAGGGCCACCACCAGCCGTCCCAATCCG
MVs/Navarra.ESP/23.17 GAACACCAGGTGGCCCCAGCATAGGACAGTCCCGACCCAGGGCCACCACCAGCCGTCCCAATCCG
MVs/Navarra.ESP/20.17 GAACACCAGGTGGCCCCAGCATAGGACAGTCCCGACCCAGGGCCACCACCAGCCGTCCCAATCCG
MVs/Valencia.ESP/47.17/47 GAACACCAGGTGGCCCCAGCATAGGACAGTCCCGACCCAGGGCCACCACCAGCCGTCCCAATCCG
MVs/Valencia.ESP/22.18/2 GAACACCAGGTGGCCCCAGCATAGGACAGTCCCGACCCAGGGCCACCACCAGCCGTCCCAATCCG
MVs/Valencia.ESP/21.18 GAACACCAGGTGGCCCCAGCATAGGACAGTCCCGACCCAGGGCCACCACCAGCCGTCCCAATCCG
MVs/Valencia.ESP/21.18/2 GAACACCAGGTGGCCCCAGCACAGGACAGTCCCGACCCAGGGCCACCACCAGCCGTCCCAATCCG
MVs/Valencia.ESP/28.28 GAACACCAGGTGGCCCCAGCATAGGACAGTCCCGACCCAGGGCCACCACCAGCCGTCCCAATCCG
MVs/Navarra.ESP/31.18 GAACACCAGGTGGCCCCAGCATAGGACAGTCCCGACCCAGGGCCACCACCAGCCGTCCCAATCCG
4599
4663
MVs/Valladolid.ESP/34.17/3 CATCCCCACCACCCCCGGGAAAGAAACCCCCAGGAGCTGGAAGGCCCTTCTCCCCCTCCCCCAAC
MVs/Cuenca.ESP/20.17 CATCCCCACCACCCCCGGGAAAGAAACCCCCAGGAGCTGGAAGGCCCTTCTCCCCCTCCCCCAAC
MVs/Navarra.ESP/18.17 CATCCCCACCACCCCCGGGAAAGAAACCCCCAGGAGCTGGAAGGCCCTTCTCCCCCTCCCCCAAC
MVs/Navarra.ESP/22.17/5 CATCCCCACCACCCCCGGGAAAGAAACCCCCAGGAGCTGGAAGGCCCTTCTCCCCCTCCCCCAAC
MVs/Navarra.ESP/23.17 CATCCCCACCACCCCCGGGAAAGAAACCCCCAGGAGCTGGAAGGCCCTTCTCCCCCTCCCCCAAC
MVs/Navarra.ESP/20.17 CATCCCCACCACCCCCGGGAAAGAAACCCCCAGGAGCTGGAAGGCCCTTCTCCCCCTCCCCCAAC
MVs/Valencia.ESP/47.17/47 CATCCCCACCACCCCCGGGAAAGAAACCCCCAGGAGCTGGAAGGCCCTTCTCCCCCTCCCCCAAC
MVs/Valencia.ESP/22.18/2 CATCCCCACCACCCCCGGGAAAGAAACCCCCAGGAGCTGGAAGGCCCTTCTCCCCCTCCCCCAAC
MVs/Valencia.ESP/21.18 CATCCCCACCACCCCCGGGAAAGAAACCCCCAGGAGCTGGAAGGCCCTTCTCCCCCTCCCCCAAC
MVs/Valencia.ESP/21.18/2 CATCCCCACCACCCCCGGGAAAGAAACCCCCAGGAGCTGGAAGGCCCTTCTCCCCCTCCCCCAAC
MVs/Valencia.ESP/28.28 CATCCCCACCACCCCCGGGAAAGAAACCCCCAGGAGCTGGAAGGCCCTTCTCCCCCTCCCCTAAC
MVs/Navarra.ESP/31.18 CATCCCCACCACCCCCGGGAAAGAAACCCCCAGGAGCTGGAAGGCCCTTCTCCCCCTCCCCCAAC

```
4664                                                                     4728
MVs/Valladolid.ESP/34.17/3   ACAAGAACCCCACAACCGAACCGCACAAGCGACCGAGGTGACCCAACCACAGGCACCCGATTCCC
MVs/Cuenca.ESP/20.17         ACAAGAACCCCACAACCGAACCGCACAAGCGACMGAGGTGACCCAACCACAGGCACCCGATTCCC
MVs/Navarra.ESP/18.17        ACAAGAACCCCACAACCGAACCGCACAAGCGACAGAGGTGACCCAACCACAGGCACCCGATTCCC
MVs/Navarra.ESP/22.17/5      ACAAGAACCCCACAACCGAACCGCACAAGCGACAGAGGTGACCCAACCACAGGCACCCGATTCCC
MVs/Navarra.ESP/23.17        ACAAGAACCCCACAACCGAACCGCACAAGCGACAGAGGTGACCCAACCACAGGCACCCGATTCCC
MVs/Navarra.ESP/20.17        ACAAGAACCCCACAACCGAACCGCACAAGCGACAGAGGTGACCCAACCACAGGCACCCGATTCCC
MVs/Valencia.ESP/47.17/47    ACAAGAACCCCACAACCGAACCGCACAAGCGACCGAGGTGACCCAACCACAGGCACCCGATTCCC
MVs/Valencia.ESP/22.18/2     ACAAGAACCCCACAACCGAACCGCACAAGCGACCGAGGTGACCCAACCACAGGCACCCGATTCCC
MVs/Valencia.ESP/21.18       ACAAGAACCCCACAACCGAACCGCACAAGCGACCGAGGTGACCCAACCACAGGCACCCGATTCCC
MVs/Valencia.ESP/21.18/2     ACAAGAACCCCACAACCGAACCGCACAAGCGACCGAGGTGACCCAACCACAGGCACCCGATTCCC
MVs/Valencia.ESP/28.28       ACAAGAACCCCACAACCGAACCGCACAAGCGACCGAGGTGACCCAACCACAGGCACCCGATTCCC
MVs/Navarra.ESP/31.18        ACAAGAACCCCACAACCGAACCGCACAAGCGACCGAGGTGACCCAACCACAGGCACTCGATTCCC

4794                                                                     4858
MVs/Valladolid.ESP/34.17/3   AGAACCCAGACCCCGGCCCACGGCACCTCGCCCCCAACCCCCGACAACCAGAGGGAGTCCCCAAC
MVs/Cuenca.ESP/20.17         AGAACCCAGACCCCGGCCCACGGCACCTCGCCCCCAACCCCCGACAACCAGAGGGAGTCCCCAAC
MVs/Navarra.ESP/18.17        AGAACCCAGACCCCGGCCCACGGCACCTCGCCCCCAACCCCCGACAACCAGAGGGAGTCCCCAAC
MVs/Navarra.ESP/22.17/5      AGAACCCAGACCCCGGCCCACGGCACCTCGCCCCCAACCCCCGACAACCAGAGGGAGTCCCCAAC
MVs/Navarra.ESP/23.17        AGAACCCAGACCCCGGCCCACGGCACCTCGCCCCCAACCCCCGACAACCAGAGGGAGTCCCCAAC
MVs/Navarra.ESP/20.17        AGAACCCAGACCCCGGCCCACGGCACCTCGCCCCCAACCCCCGACAACCAGAGGGAGTCCCCAAC
MVs/Valencia.ESP/47.17/47    AGAACCCAGACCCCAGCCCACGGCACCTCGCCCCCAACCCCCGACAACCAGAGGGAGTCCCCAAC
MVs/Valencia.ESP/22.18/2     AGAACCCAGACCCCAGCCCACGGCACCTCGCCCCCAACCCCCGACAACCAGAGGGAGTCCCCAAC
MVs/Valencia.ESP/21.18       AGAACCCAGACCCCAGCCCACGGCACCTCGCCCCCAACCCCCGACAACCAGAGGGAGTCCCCAAC
MVs/Valencia.ESP/21.18/2     AGAACCCAGACCCCAGCCCACGGCACCTCGCCCCCAACCCCCGACAACCAGAGGGAGTCCCCAAC
MVs/Valencia.ESP/28.28       AGAACCCAGACCCCAGCCCACGGCACCTCGCCCCCAACCCCCGACAACCAGAGGGAGTCCCCAAC
MVs/Navarra.ESP/31.18        AGAACCCAGACCCCGGCCCACGGCACCTCGCCCCCAACCCCCGACAACCAGGGGGAGTCCCAAAC

4859                                                                     4923
MVs/Valladolid.ESP/34.17/3   CAATCCCTCCGGCCCCCCCGGTGCCCACAGGCAGGCACACCAACCCCCAAACAGGCTCAGCACCC
MVs/Cuenca.ESP/20.17         CAATCCCTCCGGCCCCCCCGGTGCCCACAGGCAGGCACACCAACCCCCAAACAGGCCCAGCACCC
MVs/Navarra.ESP/18.17        CAATCCCTCCGGCCCCCCCGGTGCCCACAGGCAGGCACACCAACCCCCAAACAGGCCCAGCACCC
MVs/Navarra.ESP/22.17/5      CAATCCCTCCGGCCCCCCCGGTGCCCACAGGCAGGCACACCAACCCCCAAACAGGCCCAGCACCC
MVs/Navarra.ESP/23.17        CAATCCCTCCGGCCCCCCCGGTGCCCACAGGCAGGCACACCAACCCCCAAACAGGCCCAGCACCC
MVs/Navarra.ESP/20.17        CAATCCCTCCGGCCCCCCCGGTGCCCACAGGCAGGCACACCAACCCCCAAACAGGCCCAGCACCC
MVs/Valencia.ESP/47.17/47    CAATCCCTCCGGCCCCCCCGGTGCCCACAGGCAGGCACACCAACCCCCAAACAGGCCCAGCACCC
MVs/Valencia.ESP/22.18/2     CAATCCCTCCGGCCCCCCCGGTGCCCACAGGCAGGCACACCAACCCCCAAACAGGCCCAGCACCC
MVs/Valencia.ESP/21.18       CAATCCCTCCGGCCCCCCCGGTGCCCACAGGCAGGCACACCAACCCCCAAACAGGCCCAGCACCC
MVs/Valencia.ESP/21.18/2     CAATCCCTCCGGCCCCCCCGGTGCCCACAGGCAGGCACACCAACCCCCAAACAGGCCCAGCACCC
MVs/Valencia.ESP/28.28       CAATCCCTCCGGCCCCCCCGGTGCCCACAGGCAGGCACACCAACCCCCAAACAGGCCCAGCACCC
MVs/Navarra.ESP/31.18        CAATCCCTCCGGCCCCCCCGGTGCCCACAGGCAGGCACACCAACCCCCAAACAGGCCCAGCACCC

4989                                                                     5053
MVs/Valladolid.ESP/34.17/3   ACAGCGAGGGAGCCCACCCACACCACACACCACCACGGCAACCAGACCAGAACCAGACCACCCT
MVs/Cuenca.ESP/20.17         ACAGCGAGGGAGCCCACCCACACCACACACCACCACGGCAACCAGACCAGAACCAGACCACCCT
MVs/Navarra.ESP/18.17        ACAGCGAGGGAGCCCACCCACACCACACACCACCACGGCAACCAGACCAGAACCAGACCACCCT
MVs/Navarra.ESP/22.17/5      ACAGCGAGGGAGCCCACCCACACCACACACCACCACGGCAACTAGACCAGAACCAGACCACCCT
MVs/Navarra.ESP/23.17        ACAGCGAGGGAGCCCACCCACACCACACACCACCACGGCAACCAGACCAGAACCAGACCACCCT
MVs/Navarra.ESP/20.17        ACAGCGAGGGAGCCCACCCACACCACACACCACCACGGCAACCAGACCAGAACCAGACCACCCT
MVs/Valencia.ESP/47.17/47    ACAGCGAGGGAGCCCACCCACACCACACACCACCACGGCAACCAGACCAGAACCAGACCACCCT
MVs/Valencia.ESP/22.18/2     ACAGCGAGGGAGCCCACCCACACCACACACCACCACGGTAACCAGACCAGAACCAGACCACCCT
MVs/Valencia.ESP/21.18       ACAGCGAGGGAGCCCACCCACACCACACACCACCACGGTAACCAGACCAGAACCAGACCACCCT
MVs/Valencia.ESP/21.18/2     ACAGCGAGGGAGCCCACCCACACCACACACCACCACGGTAACCAGACCAGAACCAGACCACCCT
MVs/Valencia.ESP/28.28       ACAGCGAGGGAGCCCACCCACACCACACACCACCACGGTAACCAGACCAGAACCAGACCACCCT
MVs/Navarra.ESP/31.18        ACAGCGAGGGAGCCCACCCACACCACACACCACCACGGCAACCAGACCAGAACCAGACCACCCT
```

```
                          5054                                                             5118
MVs/Valladolid.ESP/34.17/3  GGGCCACCAACTCCCAGACCCGGCCACCGCCCTGCGGAAAGGGACGGCCACAACCCGCACACCCC
MVs/Cuenca.ESP/20.17        GGGCCACCAACTCCCAGACCCGGCCACCGCCCTGCGGAAAGGGACGGCCACAACCCGCACACCCC
MVs/Navarra.ESP/18.17       GGGCCACCAACTCCCAGACCCGGCCACCGCCCTGCGGAAAGGGACGGCCACAACCCGCACACCCC
MVs/Navarra.ESP/22.17/5     GGGCCACCAACTCCCAGACCCGGCCACCGCCCTGCGGAAAGGGACGGCCACAACCCGCACACCCC
MVs/Navarra.ESP/23.17       GGGCCACCAACTCCCAGACCCGGCCACCGCCCTGCGGAAAGGGACGGCCACAACCCGCACACCCC
MVs/Navarra.ESP/20.17       GGGCCACCAACTCCCAGACCCGGCCACCGCCCTGCGGAAAGGGACGGCCACAACCCGCACACCCC
MVs/Valencia.ESP/47.17/47   GGGCCACCAACTCCCAGACCCGGCCACCGCCCTGCGGAAAGGGACGGCCACAACCCGCACACCCC
MVs/Valencia.ESP/22.18/2    GGGCCACCAACTCCCAGGCCCGGCCACCGCCCTGCGGAAAGGGACGGCCACAACCCGCACACCCC
MVs/Valencia.ESP/21.18      GGGCCACCAACTCCCAGACCCGGCCACCGCCCTGCGGAAAGGGACGGCCACAACCCGCACACCCC
MVs/Valencia.ESP/21.18/2    GGGCCACCAACTCCCAGACCCGGCCACCGCCCTGCGGAAAGGGACGGCCACAACCCGCACACCCC
MVs/Valencia.ESP/28.28      GGGCCACCAACTCCCAGACCCGGCCACCGCCCTGCGGAAAGGGACGGCCACAACCCGCACACCCC
MVs/Navarra.ESP/31.18       GGGCCACCAACTCCCAGACCCGGCCACCGCCCTGCGGAAAGGGACGGCCACAACCCGCACACCCC
```

```
                          5119                                                             5183
MVs/Valladolid.ESP/34.17/3  AGCCCCGATCCGGCGGGCAGCCACCCAACCCGAACCAGCACCCAAGAGCGACCCCCGAAGGACCC
MVs/Cuenca.ESP/20.17        AGCCCCGATCCGGCGGGCAGCCACCTAACCCGAACCAGCACCCAAGAGCGACCCCCGAAGGACCC
MVs/Navarra.ESP/18.17       AGCCCCGATCCGGCGGGCAGCCACCTAACCCGAACCAGCACCCAAGAGCGACCCCCGAAGGACCC
MVs/Navarra.ESP/22.17/5     AGCCCCGATCCGGCGGGCAGCCACCTAACCCGAACCAGCACCCAAGAGCGACCCCCGAAGGACCC
MVs/Navarra.ESP/23.17       AGCCCCGATCCGGCGGGCAGCCACCTAACCCGAACCAGCACCCAAGAGCGACCCCCGAAGGACCC
MVs/Navarra.ESP/20.17       AGCCCCGATCCGGCGGGCAGCCACCTAACCCGAACCAGCACCCAAGAGCGACCCCCGAAGGACCC
MVs/Valencia.ESP/47.17/47   AGCCCCGATCCGGCGGGCAGCCACCTAACCCGAACCAGCACCCAAGAGCGACCCCCGAAGGACCC
MVs/Valencia.ESP/22.18/2    AGCCCCGATCCGGCGGGCAGCCACCTAACCCGAACCAGCACCCAAGAGCGACCCCCGAAGGACCC
MVs/Valencia.ESP/21.18      AGCCCCGATCCGGCGGGCAGCCACCTAACCCGAACCAGCACCCAAGAGCGACCCCCGAAGGACCC
MVs/Valencia.ESP/21.18/2    AGCCCCGATCCGGCGGGCAGCCACCTAACCCGAACCAGCACCCAAGAGCGACCCCCGAAGGACCC
MVs/Valencia.ESP/28.28      AGCCCCGATCCGGCGGGCAGCCACCTAACCCGAACCAGCACCCAAGAGCGACCCCCGAAGGACCC
MVs/Navarra.ESP/31.18       AGCCCCGATCCGGCGGGCAGCCACCCAACCCGAACCAGCACCCAAGAGCGACCCCCGAAGGACCC
```

```
                          5184                                                             5248
MVs/Valladolid.ESP/34.17/3  CCAAACCGCAAAGGACATCAGCATCCCACAGCCTCTCTAAGTCCGCCGGTCTCCTCCCCTTCTCG
MVs/Cuenca.ESP/20.17        CCAAACCGCAAAGGACATCAGCATCCCACAGCCTCTCTAAGTCCGCCGGTCTCCTCCCCTTCTCG
MVs/Navarra.ESP/18.17       CCAAACCGCAAAGGACATCAGCATCCCACAGCCTCTCTAAGTCCGCCGGTCTCCTCCCCTTCTCG
MVs/Navarra.ESP/22.17/5     CCAAACCGCAAAGGACATCAGCATCCCACAGCCTCTCTAAGTCCGCCGGTCTCCTCCCCTTCTCG
MVs/Navarra.ESP/23.17       CCAAACCGCAAAGGACATCAGCATCCCACAGCCTCTCTAAGTCCGCCGGTCTCCTCCCCTTCTCG
MVs/Navarra.ESP/20.17       CCAAACCGCAAAGGACATCAGCATCCCACAGCCTCTCTAAGTCCGCCGGTCTCCTCCCCTTCTCG
MVs/Valencia.ESP/47.17/47   CCAAACCGCAAAGGACATCAGCATCCCACAGCCTCTCTAAGTCCGCCGGTCTCCTCCCCTTCTCG
MVs/Valencia.ESP/22.18/2    CCAAACCGCAAAGGACATCAGCATCCCACAGCCTCTCTAAGTCCGCCGGTCTCCTCCCCTTCTCG
MVs/Valencia.ESP/21.18      CCAAACCGCAAAGGACATCAGCATCCCACAGCCTCTCTAAGTCCGCCGGTCTCCTCCCCTTCTCG
MVs/Valencia.ESP/21.18/2    CCAAACCGCAAAGGACATCAGCATCCCACAGCCTCTCTAAGTCCGCCGGTCTCCTCCCCTTCTCG
MVs/Valencia.ESP/28.28      CCAAACCGCAAAGGACATCAGCATCCCACAGCCTCTCTAAGTCCGCCGGTCTCCTCCCCTTCTCG
MVs/Navarra.ESP/31.18       CCAAACCGCAAAGGACATCAGCATCCCACAGCCTCTCCAAGTCCGCCGGTCTCCTCCCCTTCTCG
```
