# Supplementary material for: Utility of MF-non coding region for measles molecular surveillance during post-elimination phase, Spain, 2017–2020
Source: Front Microbiol. 2023 May 22;14:1143933. doi: 10.3389/fmicb.2023.1143933 (PMC10240958; doi:10.3389/fmicb.2023.1143933)
Supplement: Supplementary Figure S2 — Alignment of the MF-NCR region for the 15 sets of identical MF-NCR sequences found in D8. Regions without substitutions are not shown in the sake of place. [file Data_Sheet_2.zip › Figure S2.pdf]

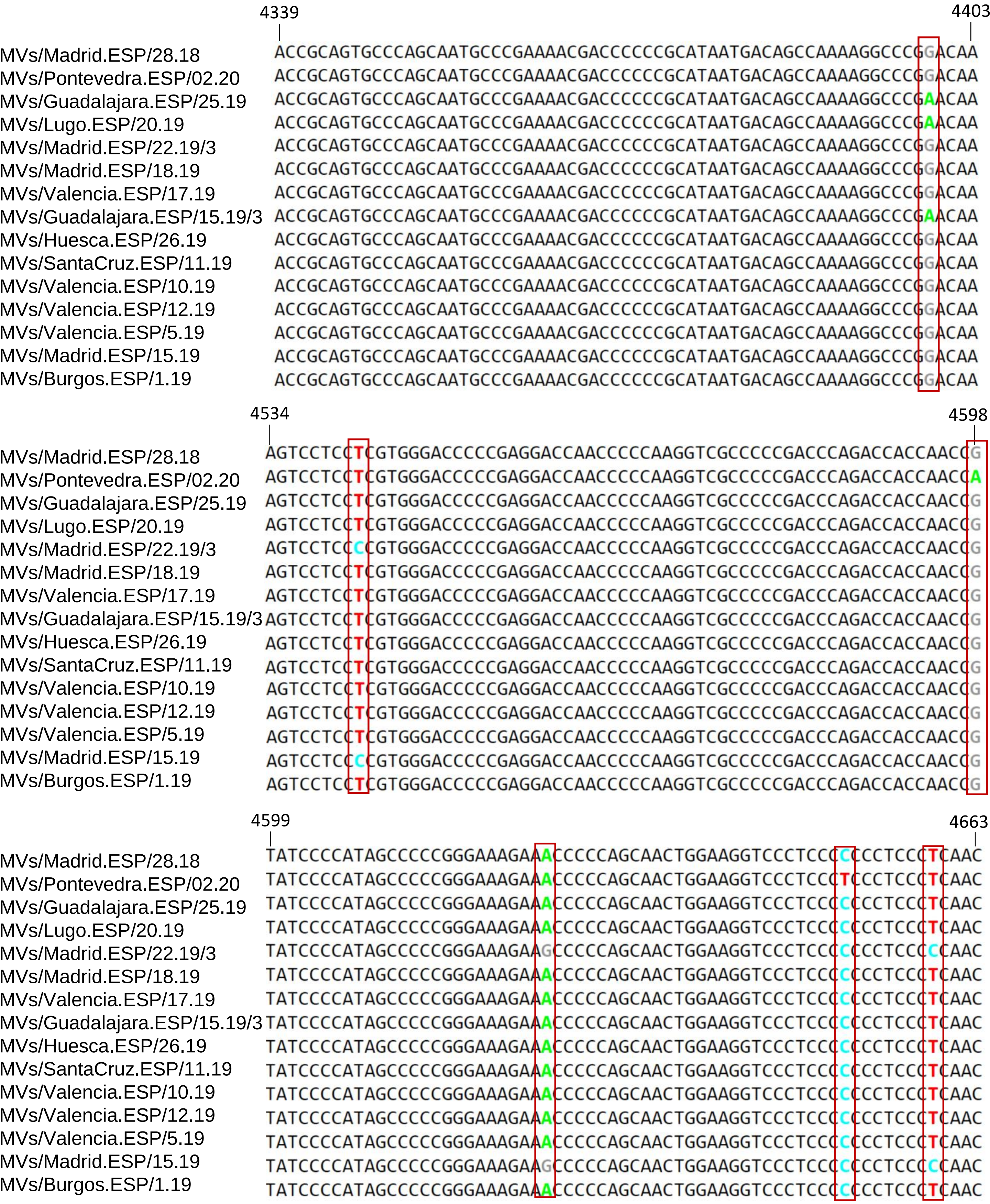
4339
4403
MVs/Madrid.ESP/28.18 ACCGCAGTGCCCAGCAATGCCCGAAAACGACCCCCCGCATAATGACAGCCAAAAGGCCCGGACAA
MVs/Pontevedra.ESP/02.20 ACCGCAGTGCCCAGCAATGCCCGAAAACGACCCCCCGCATAATGACAGCCAAAAGGCCCGGACAA
MVs/Guadalajara.ESP/25.19 ACCGCAGTGCCCAGCAATGCCCGAAAACGACCCCCCGCATAATGACAGCCAAAAGGCCCGAACAA
MVs/Lugo.ESP/20.19 ACCGCAGTGCCCAGCAATGCCCGAAAACGACCCCCCGCATAATGACAGCCAAAAGGCCCGAACAA
MVs/Madrid.ESP/22.19/3 ACCGCAGTGCCCAGCAATGCCCGAAAACGACCCCCCGCATAATGACAGCCAAAAGGCCCGGACAA
MVs/Madrid.ESP/18.19 ACCGCAGTGCCCAGCAATGCCCGAAAACGACCCCCCGCATAATGACAGCCAAAAGGCCCGGACAA
MVs/Valencia.ESP/17.19 ACCGCAGTGCCCAGCAATGCCCGAAAACGACCCCCCGCATAATGACAGCCAAAAGGCCCGGACAA
MVs/Guadalajara.ESP/15.19/3 ACCGCAGTGCCCAGCAATGCCCGAAAACGACCCCCCGCATAATGACAGCCAAAAGGCCCGAACAA
MVs/Huesca.ESP/26.19 ACCGCAGTGCCCAGCAATGCCCGAAAACGACCCCCCGCATAATGACAGCCAAAAGGCCCGGACAA
MVs/SantaCruz.ESP/11.19 ACCGCAGTGCCCAGCAATGCCCGAAAACGACCCCCCGCATAATGACAGCCAAAAGGCCCGGACAA
MVs/Valencia.ESP/10.19 ACCGCAGTGCCCAGCAATGCCCGAAAACGACCCCCCGCATAATGACAGCCAAAAGGCCCGGACAA
MVs/Valencia.ESP/12.19 ACCGCAGTGCCCAGCAATGCCCGAAAACGACCCCCCGCATAATGACAGCCAAAAGGCCCGGACAA
MVs/Valencia.ESP/5.19 ACCGCAGTGCCCAGCAATGCCCGAAAACGACCCCCCGCATAATGACAGCCAAAAGGCCCGGACAA
MVs/Madrid.ESP/15.19 ACCGCAGTGCCCAGCAATGCCCGAAAACGACCCCCCGCATAATGACAGCCAAAAGGCCCGGACAA
MVs/Burgos.ESP/1.19 ACCGCAGTGCCCAGCAATGCCCGAAAACGACCCCCCGCATAATGACAGCCAAAAGGCCCGGACAA
4534
4598
MVs/Madrid.ESP/28.18 AGTCCTCCTCGTGGGACCCCCGAGGACCAACCCCCAAGGTCGCCCCCGACCCAGACCACCAACCG
MVs/Pontevedra.ESP/02.20 AGTCCTCCTCGTGGGACCCCCGAGGACCAACCCCCAAGGTCGCCCCCGACCCAGACCACCAACCA
MVs/Guadalajara.ESP/25.19 AGTCCTCCTCGTGGGACCCCCGAGGACCAACCCCCAAGGTCGCCCCCGACCCAGACCACCAACCG
MVs/Lugo.ESP/20.19 AGTCCTCCTCGTGGGACCCCCGAGGACCAACCCCCAAGGTCGCCCCCGACCCAGACCACCAACCG
MVs/Madrid.ESP/22.19/3 AGTCCTCCCCGTGGGACCCCCGAGGACCAACCCCCAAGGTCGCCCCCGACCCAGACCACCAACCG
MVs/Madrid.ESP/18.19 AGTCCTCCTCGTGGGACCCCCGAGGACCAACCCCCAAGGTCGCCCCCGACCCAGACCACCAACCG
MVs/Valencia.ESP/17.19 AGTCCTCCTCGTGGGACCCCCGAGGACCAACCCCCAAGGTCGCCCCCGACCCAGACCACCAACCG
MVs/Guadalajara.ESP/15.19/3 AGTCCTCCTCGTGGGACCCCCGAGGACCAACCCCCAAGGTCGCCCCCGACCCAGACCACCAACCG
MVs/Huesca.ESP/26.19 AGTCCTCCTCGTGGGACCCCCGAGGACCAACCCCCAAGGTCGCCCCCGACCCAGACCACCAACCG
MVs/SantaCruz.ESP/11.19 AGTCCTCCTCGTGGGACCCCCGAGGACCAACCCCCAAGGTCGCCCCCGACCCAGACCACCAACCG
MVs/Valencia.ESP/10.19 AGTCCTCCTCGTGGGACCCCCGAGGACCAACCCCCAAGGTCGCCCCCGACCCAGACCACCAACCG
MVs/Valencia.ESP/12.19 AGTCCTCCTCGTGGGACCCCCGAGGACCAACCCCCAAGGTCGCCCCCGACCCAGACCACCAACCG
MVs/Valencia.ESP/5.19 AGTCCTCCTCGTGGGACCCCCGAGGACCAACCCCCAAGGTCGCCCCCGACCCAGACCACCAACCG
MVs/Madrid.ESP/15.19 AGTCCTCCCCGTGGGACCCCCGAGGACCAACCCCCAAGGTCGCCCCCGACCCAGACCACCAACCG
MVs/Burgos.ESP/1.19 AGTCCTCCTCGTGGGACCCCCGAGGACCAACCCCCAAGGTCGCCCCCGACCCAGACCACCAACCG
4599
4663
MVs/Madrid.ESP/28.18 TATCCCCATAGCCCCCGGGAAAGAAACCCCCAGCAACTGGAAGGTCCCTCCCCCCCTCCCTCAAC
MVs/Pontevedra.ESP/02.20 TATCCCCATAGCCCCCGGGAAAGAAACCCCCAGCAACTGGAAGGTCCCTCCCTCCCTCCCTCAAC
MVs/Guadalajara.ESP/25.19 TATCCCCATAGCCCCCGGGAAAGAAACCCCCAGCAACTGGAAGGTCCCTCCCCCCCTCCCTCAAC
MVs/Lugo.ESP/20.19 TATCCCCATAGCCCCCGGGAAAGAAACCCCCAGCAACTGGAAGGTCCCTCCCCCCCTCCCTCAAC
MVs/Madrid.ESP/22.19/3 TATCCCCATAGCCCCCGGGAAAGAAGCCCCCAGCAACTGGAAGGTCCCTCCCCCCCTCCCCCAAC
MVs/Madrid.ESP/18.19 TATCCCCATAGCCCCCGGGAAAGAAACCCCCAGCAACTGGAAGGTCCCTCCCCCCCTCCCTCAAC
MVs/Valencia.ESP/17.19 TATCCCCATAGCCCCCGGGAAAGAAACCCCCAGCAACTGGAAGGTCCCTCCCCCCCTCCCTCAAC
MVs/Guadalajara.ESP/15.19/3 TATCCCCATAGCCCCCGGGAAAGAAACCCCCAGCAACTGGAAGGTCCCTCCCCCCCTCCCTCAAC
MVs/Huesca.ESP/26.19 TATCCCCATAGCCCCCGGGAAAGAAACCCCCAGCAACTGGAAGGTCCCTCCCCCCCTCCCTCAAC
MVs/SantaCruz.ESP/11.19 TATCCCCATAGCCCCCGGGAAAGAAACCCCCAGCAACTGGAAGGTCCCTCCCCCCCTCCCTCAAC
MVs/Valencia.ESP/10.19 TATCCCCATAGCCCCCGGGAAAGAAACCCCCAGCAACTGGAAGGTCCCTCCCCCCCTCCCTCAAC
MVs/Valencia.ESP/12.19 TATCCCCATAGCCCCCGGGAAAGAAACCCCCAGCAACTGGAAGGTCCCTCCCCCCCTCCCTCAAC
MVs/Valencia.ESP/5.19 TATCCCCATAGCCCCCGGGAAAGAAACCCCCAGCAACTGGAAGGTCCCTCCCCCCCTCCCTCAAC
MVs/Madrid.ESP/15.19 TATCCCCATAGCCCCCGGGAAAGAAGCCCCCAGCAACTGGAAGGTCCCTCCCCCCCTCCCCCAAC
MVs/Burgos.ESP/1.19 TATCCCCATAGCCCCCGGGAAAGAAACCCCCAGCAACTGGAAGGTCCCTCCCCCCCTCCCTCAAC

Strain	4794–4858	4859–4923	4924–4988
MVs/Madrid.ESP/28.18	AGAACCCAGACCCCGGCCCACGACGCCGCGCCCCCATCCCCCGACAACCAGAGAGAGCCCCCAAC	CAATCCCGCCGGCTCCCCCGGCGCCCACAGGCAGGCACACCAACCCCCGAACAGACCCGGCACCC	AGCCACCGACAGTCCAAGACGGGGGGCCCCCCCAAAAAAAGGCCCCCAGGGGCCGACAGCCAGC
MVs/Pontevedra.ESP/02.20	AGAACCCAGACCCCGGCCCACGACGCCGCGCCCCCATCCCCCGACAACCAGAGAGAGCCCCCAAC	CAGTCCCGCCGGCTCCCCCGGCGCCCACAGGCAGGCACACCAACCCCCGAACAGACCCGGCACCC	AGCCACCGACAGTCCAAGACGGGGGGCCCCCCCAAAAAAAGGCCCCCAGGGGCCGACAGCCAGC
MVs/Guadalajara.ESP/25.19	AGAACCCAGACCCCGGCCCACGACGCCGCGCCCCCATCCCCCGACAACCAGAGAGAGCCCCCAAC	CAATCCCGCCGGTTCCCCCGGCGCCCACAGGCAGGCACACCAACCCCCGAACAGACCCGGCACCC	AGCCACCGACAGTCCAAGACGGGGGGCCCCCCCAAAAAAAGGCCCCCAGGGGCCGACAGCCAGC
MVs/Lugo.ESP/20.19	AGAACCCAGACCCCGGCCCACGACGCCGCGCCCCCATCCCCCGACAACCAGAGAGAGCCCCCAAC	CAATCCCGCCGGCTCCCCCGGCGCCCACAGGCAGGCACACCAACCCCCGAACAGACCCGGCACCC	AGCCACCGACAGTCCAAGACGGGGGGCCCCCCCAAAAAAAGGCCCCCAGGGGCCGACAGCCAGC
MVs/Madrid.ESP/22.19/3	AGAACCCAGACCCCGGCCCACGACGCCGCGCCCCCATCCCCCGACAACCAGAGAGAGCCCCCAAC	CAATCCCGCCGGCTACCCCGGCGCCCACAGGCAGGCACACCAACCCCCGAACAGACCCGGCACCC	AGCCACCGACAGTCCAAGACGGGGGGCCCCCCCAAAAAAAGGCCCCCAGGGGCCGACAGCCAGC
MVs/Madrid.ESP/18.19	AGAACCCAGACCCCGGCCCACGACGCCGCGCCCCCATCCCCCGACAACCAGAGAGAGCCCCCAAC	CAATCCCGCCGGCTCCCCCGGCGCCCACAGGCAGGCACACCAACCCCCGAACAGACCCGGCACCC	AGCCACCGACAGTCCAAGACGGGGGGCCCCCCCAAAAAAAGGCCCCCAGGGGCCGACAGCCAGC
MVs/Valencia.ESP/17.19	AGAACCCAGACCCCGGCCCACGACGCCGCGCCCCCATCCCCCGACAACCAGAGAGAGCCCCCAAC	CAATCCCGCCGGCTCCCCCGGCGCCCACAGGCAGGCACACCAACCCCCGAACAGACCCGGCACCC	AGCCACCGACAGTCCAAGACGGGGGGCCCCCCCAAAAAAAGGCCCCCAGGGGCCGACAGCCAGC
MVs/Guadalajara.ESP/15.19/3	AGAACCCAGACCCCGGCCCACGACGCCGCGCCCCCATCCCCCGACAACCAGAGAGAGCCCCCAAC	CAATCCCGCCGGCTCCCCCGGCGCCCACAGGCAGGCACACCAACCCCCGAACAGACCCGGCACCC	AGCCACCGACAGTCCAAGACGGGGGGCCCCCCCAAAAAAAGGCCCCCAGGGGCCGACAGCCAGC
MVs/Huesca.ESP/26.19	AGAACCCAGACCCCGGCCCACGACGCCGCGCCCCCATCCCCCGACAACCAGAGAGAGCCCCCAAC	CAGTCCCGCCGGCTCCCCCGGCGCCCACAGGCAGGCACACCAACCCCCGAACAGACCCGGCACCC	AGCCACCGACAGTCCAAGACGGGGGGCCCCCCCAAAAAAAGGCCCCCAGGGGCCGACAGCCAGC
MVs/SantaCruz.ESP/11.19	AGAACCCAGACCCCGGCCCACGACGCCGCGCCCCCATCCCCCGACAACCAGAGAGAGCCCCCAAC	CAATCCCGCCGGCTCCCCCGGCGCCCACAGGCAGGCACACCAACCCCCGAACAGACCCGGCACCC	AGCCACCGACAGTCCAAGACGGGGGGCCCCCCCAAAAAAAAGCCCCCAGGGGCCGACAGCCAGC
MVs/Valencia.ESP/10.19	AGAACCCAGACCCCGGCCCACGACGCCGCGCCCCCATCCCCCGACAACCAGAGAGAGCCCCCAAC	CAATCCCGCCGGCTCCCCCGGCGCCCACAGGCAGGCACACCAACCCCCGAACAGACCCGGCACCC	AGCCACCGACAGTCCAAGACGGGGGGTCCCCCCAAAAAAAGGCCCCCAGGGGCCGACAGCCAGC
MVs/Valencia.ESP/12.19	AGAACTCAGACCCCGGCCCACGACGCCGCGCCCCCATCCCTCGACAACCAGAGAGAGCCCCCAAC	CAATCCCGCCGGCTCCCCCGGCGCCCACAGGCAGGCACACCAACCCCCGAACAGACCCGGCACCC	AGCCACCGACAGTCCAAGACGGGGGGCCCCCCCAAAAAAAAGCCCCCAGGGGCCGACAGCCAGC
MVs/Valencia.ESP/5.19	AGAACCCAGACCCCGGCCCACGACGCCGCGCCCCCATCCCCCGACAACCAGAGAGAGCCCCCAAC	CAATCCCGCCGGCTCCCCCGGCGCCCACAGGCAGGCACACCAACCCCCGAACAGACCCGGCACCC	AGCCACCGACAGTCCAAGACGGGGGGCCCCCCCAAAAAAAGGCCCCCAGGGGCCGACAGCCAGC
MVs/Madrid.ESP/15.19	AGAACCCAGACCCCGGCCCACGACGCCGCGCCCCCATCCCCCGACAACCAGAGAGAGCCCCCAAC	CAATCCCGCCGGCTCCCCCGGCGCCCACAGGCAGGCACACCAACCCCCGAACAGACCCGGCACCC	AGCCACCGACAGTCCAAGACGGGGGGCCCCCCCAAAAAAAGGCCCCCAGGGGCCGACAGCCAGC
MVs/Burgos.ESP/1.19	AGAACCCAGACCCCGGCCCACGACGCCGCGCCCCCATCCCCCGACAGCCAGAGAGAGCCCCCAAC	CAATCCCGCCGGCTCCCCCGGCGCCCACAGGCAGGCACACCAACCCCCGAACAGACCCGGCACCC	AGCCACCGACAGTCCAAGACGGGGGGCCCCCCCAAAAAAAGGCCCCCAGGGGCCGACAGCCAGC

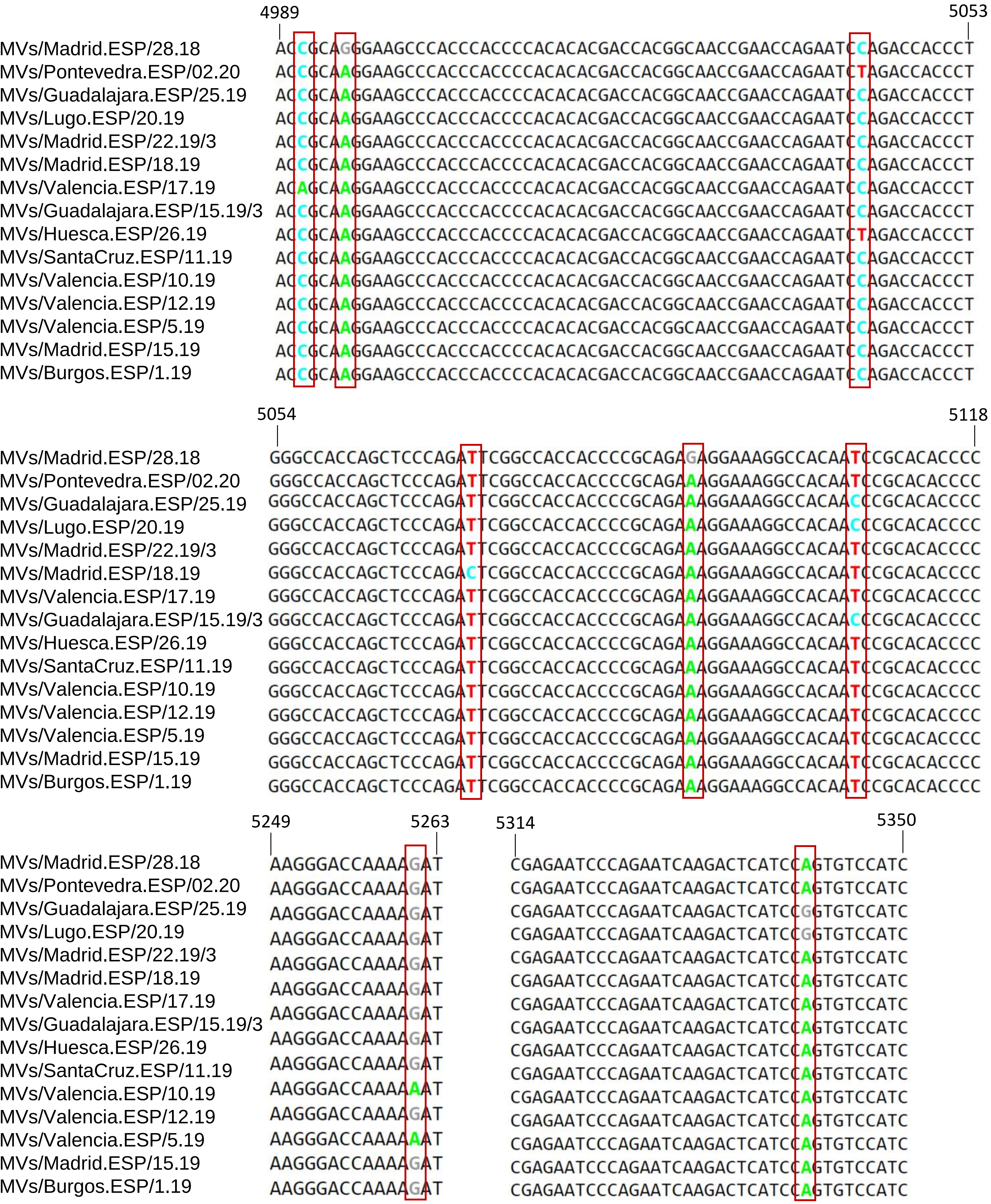

4989
5053
MVs/Madrid.ESP/28.18 ACCGCAGGGAAGCCCACCCACCCCACACACGACCACGGCAACCGAACCAGAATCCAGACCACCCT
MVs/Pontevedra.ESP/02.20 ACCGCAAGGAAGCCCACCCACCCCACACACGACCACGGCAACCGAACCAGAATCTAGACCACCCT
MVs/Guadalajara.ESP/25.19 ACCGCAAGGAAGCCCACCCACCCCACACACGACCACGGCAACCGAACCAGAATCCAGACCACCCT
MVs/Lugo.ESP/20.19 ACCGCAAGGAAGCCCACCCACCCCACACACGACCACGGCAACCGAACCAGAATCCAGACCACCCT
MVs/Madrid.ESP/22.19/3 ACCGCAAGGAAGCCCACCCACCCCACACACGACCACGGCAACCGAACCAGAATCCAGACCACCCT
MVs/Madrid.ESP/18.19 ACCGCAAGGAAGCCCACCCACCCCACACACGACCACGGCAACCGAACCAGAATCCAGACCACCCT
MVs/Valencia.ESP/17.19 ACAGCAAGGAAGCCCACCCACCCCACACACGACCACGGCAACCGAACCAGAATCCAGACCACCCT
MVs/Guadalajara.ESP/15.19/3 ACCGCAAGGAAGCCCACCCACCCCACACACGACCACGGCAACCGAACCAGAATCCAGACCACCCT
MVs/Huesca.ESP/26.19 ACCGCAAGGAAGCCCACCCACCCCACACACGACCACGGCAACCGAACCAGAATCTAGACCACCCT
MVs/SantaCruz.ESP/11.19 ACCGCAAGGAAGCCCACCCACCCCACACACGACCACGGCAACCGAACCAGAATCCAGACCACCCT
MVs/Valencia.ESP/10.19 ACCGCAAGGAAGCCCACCCACCCCACACACGACCACGGCAACCGAACCAGAATCCAGACCACCCT
MVs/Valencia.ESP/12.19 ACCGCAAGGAAGCCCACCCACCCCACACACGACCACGGCAACCGAACCAGAATCCAGACCACCCT
MVs/Valencia.ESP/5.19 ACCGCAAGGAAGCCCACCCACCCCACACACGACCACGGCAACCGAACCAGAATCCAGACCACCCT
MVs/Madrid.ESP/15.19 ACCGCAAGGAAGCCCACCCACCCCACACACGACCACGGCAACCGAACCAGAATCCAGACCACCCT
MVs/Burgos.ESP/1.19 ACCGCAAGGAAGCCCACCCACCCCACACACGACCACGGCAACCGAACCAGAATCCAGACCACCCT
5054
5118
MVs/Madrid.ESP/28.18 GGGCCACCAGCTCCCAGATTCGGCCACCACCCCGCAGAGAGGAAAGGCCACAATCCGCACACCCC
MVs/Pontevedra.ESP/02.20 GGGCCACCAGCTCCCAGATTCGGCCACCACCCCGCAGAAAGGAAAGGCCACAATCCGCACACCCC
MVs/Guadalajara.ESP/25.19 GGGCCACCAGCTCCCAGATTCGGCCACCACCCCGCAGAAAGGAAAGGCCACAACCCGCACACCCC
MVs/Lugo.ESP/20.19 GGGCCACCAGCTCCCAGATTCGGCCACCACCCCGCAGAAAGGAAAGGCCACAACCCGCACACCCC
MVs/Madrid.ESP/22.19/3 GGGCCACCAGCTCCCAGATTCGGCCACCACCCCGCAGAAAGGAAAGGCCACAATCCGCACACCCC
MVs/Madrid.ESP/18.19 GGGCCACCAGCTCCCAGACTCGGCCACCACCCCGCAGAAAGGAAAGGCCACAATCCGCACACCCC
MVs/Valencia.ESP/17.19 GGGCCACCAGCTCCCAGATTCGGCCACCACCCCGCAGAAAGGAAAGGCCACAATCCGCACACCCC
MVs/Guadalajara.ESP/15.19/3 GGGCCACCAGCTCCCAGATTCGGCCACCACCCCGCAGAAAGGAAAGGCCACAACCCGCACACCCC
MVs/Huesca.ESP/26.19 GGGCCACCAGCTCCCAGATTCGGCCACCACCCCGCAGAAAGGAAAGGCCACAATCCGCACACCCC
MVs/SantaCruz.ESP/11.19 GGGCCACCAGCTCCCAGATTCGGCCACCACCCCGCAGAAAGGAAAGGCCACAATCCGCACACCCC
MVs/Valencia.ESP/10.19 GGGCCACCAGCTCCCAGATTCGGCCACCACCCCGCAGAAAGGAAAGGCCACAATCCGCACACCCC
MVs/Valencia.ESP/12.19 GGGCCACCAGCTCCCAGATTCGGCCACCACCCCGCAGAAAGGAAAGGCCACAATCCGCACACCCC
MVs/Valencia.ESP/5.19 GGGCCACCAGCTCCCAGATTCGGCCACCACCCCGCAGAAAGGAAAGGCCACAATCCGCACACCCC
MVs/Madrid.ESP/15.19 GGGCCACCAGCTCCCAGATTCGGCCACCACCCCGCAGAAAGGAAAGGCCACAATCCGCACACCCC
MVs/Burgos.ESP/1.19 GGGCCACCAGCTCCCAGATTCGGCCACCACCCCGCAGAAAGGAAAGGCCACAATCCGCACACCCC
5249
5263
5314
5350
MVs/Madrid.ESP/28.18 AAGGGACCAAAAGAT CGAGAATCCCAGAATCAAGACTCATCCAGTGTCCATC
MVs/Pontevedra.ESP/02.20 AAGGGACCAAAAGAT CGAGAATCCCAGAATCAAGACTCATCCAGTGTCCATC
MVs/Guadalajara.ESP/25.19 AAGGGACCAAAAGAT CGAGAATCCCAGAATCAAGACTCATCCGGTGTCCATC
MVs/Lugo.ESP/20.19 AAGGGACCAAAAGAT CGAGAATCCCAGAATCAAGACTCATCCGGTGTCCATC
MVs/Madrid.ESP/22.19/3 AAGGGACCAAAAGAT CGAGAATCCCAGAATCAAGACTCATCCAGTGTCCATC
MVs/Madrid.ESP/18.19 AAGGGACCAAAAGAT CGAGAATCCCAGAATCAAGACTCATCCAGTGTCCATC
MVs/Valencia.ESP/17.19 AAGGGACCAAAAGAT CGAGAATCCCAGAATCAAGACTCATCCAGTGTCCATC
MVs/Guadalajara.ESP/15.19/3 AAGGGACCAAAAGAT CGAGAATCCCAGAATCAAGACTCATCCAGTGTCCATC
MVs/Huesca.ESP/26.19 AAGGGACCAAAAGAT CGAGAATCCCAGAATCAAGACTCATCCAGTGTCCATC
MVs/SantaCruz.ESP/11.19 AAGGGACCAAAAGAT CGAGAATCCCAGAATCAAGACTCATCCAGTGTCCATC
MVs/Valencia.ESP/10.19 AAGGGACCAAAAAAT CGAGAATCCCAGAATCAAGACTCATCCAGTGTCCATC
MVs/Valencia.ESP/12.19 AAGGGACCAAAAGAT CGAGAATCCCAGAATCAAGACTCATCCAGTGTCCATC
MVs/Valencia.ESP/5.19 AAGGGACCAAAAAAT CGAGAATCCCAGAATCAAGACTCATCCAGTGTCCATC
MVs/Madrid.ESP/15.19 AAGGGACCAAAAGAT CGAGAATCCCAGAATCAAGACTCATCCAGTGTCCATC
MVs/Burgos.ESP/1.19 AAGGGACCAAAAGAT CGAGAATCCCAGAATCAAGACTCATCCAGTGTCCATC
